# Assessing the Acute and Chronic Effects of Palladium on *Daphnia magna* and the Influence of Natural Organic Matter

**DOI:** 10.1002/jat.4854

**Published:** 2025-07-10

**Authors:** Rania Boukhari, Dominic E. Ponton, Maikel Rosabal, Kristin K. Mueller, Marc Amyot

**Affiliations:** ^1^ GRIL, Département des Sciences Biologiques Université de Montréal Montreal Quebec Canada; ^2^ EcotoQ, Centre de Recherche en Écotoxicologie du Québec Quebec Quebec Canada; ^3^ GRIL, Département des Sciences Biologiques Université du Québec à Montréal (UQAM) Montreal Quebec Canada; ^4^ Centre d'Expertise en Analyse Environnementale du Québec Ministère de l'Environnement et de la Lutte aux Changements Climatiques Quebec Quebec Canada

**Keywords:** dissolved organic carbon, lethal, platinum group element, sublethal, toxicity

## Abstract

In recent decades, rising industrial demand for palladium (Pd), driven by its unique properties and affordability compared to other noble metals, has increased its environmental release into aquatic systems. This highlights the need to assess its effects on organisms, given the lack of standardized toxicity studies and regulations for this element. This study examines the acute and chronic impacts of Pd exposure on *D. magna* . Acute lethality was assessed over 48 h at measured Pd concentrations ranging from 2 to 110 μg/L, yielding an LC_50_ of 52 ± 2 μg/L and an LC_10_ of 33 ± 3 μg/L. The impact of natural organic matter from the Suwannee River on Pd lethality and bioaccumulation was also examined. No mortality occurred at DOC concentrations of 1, 2, 5, and 8 mg C/L, in contrast with the results obtained at 0.8 mg C/L, which resulted in an LC_50_ of 74 ± 1 μg/L. Pd bioaccumulation decreased significantly with increasing DOC concentrations compared to controls. After 15 days of chronic exposure, offspring viability significantly declined, with an EC_50_ of 1 ± 0.1 μg/L, alongside reductions in total broods per *D. magna* (EC_50_: 7 ± 2 μg/L). Parental dry weight also decreased significantly, though the timing of the first brood and weight‐normalized oxygen consumption rates remained unaffected across treatments. Parental survival was notably affected, with an LC_50_ value of 14 ± 2 μg/L. These results emphasize Pd's potential for both lethal and sublethal effects, highlighting the need for environmental standards to protect aquatic life.

## Abstract 2

This study examines acute and chronic impacts of Pd exposure on *Daphnia magna* . Acute lethality yielded an LC_50_ of 52 ± 2 μg/L and an LC_10_ of 33 ± 3 μg/L. Pd toxicity and bioaccumulation decreased with increasing dissolved organic carbon concentrations. Chronic exposure led to a decline in offspring viability and total brood size. These results emphasize Pd's potential for lethal and sublethal effects, highlighting the need for environmental standards to protect aquatic life.

## 1 Introduction

Palladium (Pd) is a precious and rare metal belonging to the platinum group elements (PGEs), which also includes platinum (Pt), rhodium (Rh), iridium (Ir), ruthenium (Ru), and osmium (Os). These metals exhibit exceptional physical and chemical properties such as resistance to corrosion and oxidation, high melting points, and strong catalytic activity that support their extensive use in industrial and technological applications, including catalytic converters, electronics, and jewelry (Tang et al. 2023). Canada ranks fourth globally in Pd production with 16 tons in 2023 (US Geological Survey 2024) and classifies PGEs as critical and strategic metals (Government of Canada 2024).

Although the concentrations of PGEs in the upper continental crust are on the order of 0.01–0.5 μg/g (Rudnick and Gao 2014), anthropogenic emissions, mainly from the automotive sector, have increased their environmental levels. Pd, a key component of catalytic converters, is the most emitted and bioavailable PGE (Wei et al. 2019; Fortin et al. 2011). Studies have documented PGE contamination in soils (Cicchella et al. 2003; Matodzi et al. 2020; Savignan et al. 2021), road dust (Spada et al. 2012; Wiseman et al. 2016; Ladonin 2018), sediments (Rauch et al. 2004; Whiteley and Murray 2005), and freshwater (Wood and Vlassopoulos 1990; Odiyo et al. 2005; Soyol‐Erdene and Huh 2012). In freshwater systems, Pd concentrations are generally very low, often below detection limits (~1 ng/L) in both urban and remote regions of Quebec, Canada (Bluteau et al. 2025). In contrast, slightly higher levels have been detected in the Estuary and Gulf of St. Lawrence, with dissolved Pd ranging from 0.085 to 0.29 ng/L (Dang et al. 2024). In biota, Pd bioaccumulation can reach notable levels; for instance, concentrations in *Asellus aquaticus* from an urban river in Gothenburg, Sweden, reached 155 ± 73 ng/g, exceeding those of Pt and Rh (Moldovan et al. 2001). However, accurate Pd quantification in biological tissues remains analytically challenging due to spectral interferences (e.g., CuAr^+^ and YO^+^ on 105Pd), which may lead to overestimation unless advanced analytical corrections are applied (Bluteau et al. 2025). Despite low environmental concentrations, Pd remains bioavailable and potentially toxic. Its complexation with organic and inorganic ligands, as a class B metal, further complicates assessments of its bioavailability and environmental impact.

Dissolved organic carbon (DOC) in natural waters serves as a key metal‐binding ligand, greatly affecting metal bioavailability and bioaccumulation. In *Dreissena polymorpha* , the effect of organic matter on Pd internalization appears to vary across studies. Sures and Zimmermann (2007) reported decreased Pd internalization in the presence of humic substances compared to tap water, suggesting a protective role of DOC. In contrast, Zimmermann et al. (2002) observed increased uptake under similar conditions. This discrepancy underscores uncertainties in PGE absorption studies involving dissolved organic matter (DOM).

The accumulation of these metals in living organisms can cause adverse effects. Studies have indicated that Pd exhibits acute toxicity to certain aquatic organisms, such as zebrafish, with an LC_50_ of 293 μg/L after 96 h of exposure (Chen et al. 2015). Additionally, research on *D. magna* demonstrated that Pd was the most toxic of the PGEs, with an LC_50_ of 14 μg/L, compared to Pt and Rh, which had LC_50_ values of 157 and 57 μg/L, respectively (Zimmermann et al. 2017). Sublethal effects such as reduced growth, reduced heart rate, and abnormal behavior have also been reported for Pd (Fujiwara et al. 2008; Sawasdee and Köhler 2010; Wren and Gagnon 2014). However, many studies do not fully comply with OECD (Organisation for Economic Co‐operation and Development)/ISO (International Organization for Standardization) guidelines, often lacking measured exposure concentrations and bioaccumulation data. Also, they rarely consider DOC complexation. Evaluating Pd toxicity thus requires rigorous experimental approaches and chemical speciation analysis to ensure accurate risk assessments.

Physiological changes in organisms can also indicate the toxicity of a substance, with the measurement of metabolic rate being a key assessment method (Barbieri 2009). Respiration is often used to assess metabolic rate, linking oxygen consumption to energy production (Nikinmaa et al. 2019). Respirometry analyzes have already been used to assess environmental stress in animals, particularly in invertebrates (Zhang et al. 2014) and fish (Witeska et al. 2010; Cocherell et al. 2011; de Medeiros et al. 2020). However, data on the effects of PGEs on the respiration of organisms are rare, requiring more in‐depth research in this area.

Overall, data on the toxicity of PGEs remain limited in the literature, hampering the development of solid species sensitivity distribution (SSD) curves commonly used to derive governmental guidelines. This is especially the case for chronic toxicity data. This study employs *D. magna* , a freshwater crustacean that is widely recognized as a standard model in ecotoxicology (Ebert 2005). This study examines the effects of acute and chronic Pd exposure on *D. magna* , focusing on critical endpoints such as mortality, reproduction, bioaccumulation, and respiratory function. Additionally, it examines the role of DOC in modulating Pd bioaccumulation and toxicity. Acute Pd exposure is expected to induce pronounced toxic responses in a concentration‐dependent manner, whereas DOC may reduce Pd's bioaccumulation and mitigate its toxic effects. Chronic exposure is anticipated to result in sublethal effects, including impaired reproductive performance and disruptions in respiratory metabolism, which could disturb energy homeostasis and ultimately compromise the organism's physiological integrity and overall fitness.

## 2 Material and Methods

### 2.1 Test Organism

The experiments were conducted using the model organism *D. magna* , provided by Environment and Climate Change Canada (ECCC) in Vancouver. The daphnids were cultured under controlled laboratory conditions at Université de Montréal, adhering to the protocol established by the Centre d'Expertise en Analyse Environnementale du Québec (CEAEQ) and the Organisation for Economic Co‐operation and Development (OECD) guideline (OECD 2004; CEAEQ 2021). Standard reference water was prepared using a composition of CaCl_2_ x 2H_2_O (0.195 g/L), NaHCO_3_ (0.0648 g/L), KCl (0.0058 g/L), and MgSO_4_ x 7H_2_O (0.0822 g/L), all dissolved in deionized water. M4 medium was initially used to maintain good culture conditions, promoting high reproductive output and minimizing mortality (Elendt and Bias 1990). However, as an alternative to M4 medium, a commercially sourced YCT diet (Aquatic Research Organisms)—a blend of yeast, CEROPHYLL, and trout chow—was administered at a concentration of 0.5 μL/mL of culture water, three times per week (Cho et al. 2022). This YCT diet, commonly used in metal toxicity testing, was provided particularly before and during chronic exposures to ensure adequate nutrition and support reproductive consistency.

The daphnids were maintained in 30‐L glass aquaria, with a population density of 10–15 individuals per liter. The water was continuously aerated to maintain approximately 80% oxygen saturation. A 16‐h light/8‐h dark cycle was applied, and the laboratory temperature ranged from 20°C to 24°C. Key water parameters, such as pH and dissolved oxygen, were monitored weekly during routine water changes to ensure optimal water quality in the culture. The daphnids were fed daily with 7.2 × 10^5^ cells from a concentrated algae solution, corresponding to 4.8 × 10^4^ cells/mL for each 15 L of aquarium water. The algae culture, consisting of *Raphidocelis subcapitata* (strain UTEX 1648) sourced from the University of Texas at Austin, was prepared in a 10‐L volume and grown until reaching a cell density of 24 × 10^6^ cells/mL. The algae were cultured under continuous light and aeration in a growth chamber at a controlled temperature of 24°C ± 2°C.

### 2.2 Exposure Conditions

The exposure media was spiked with a standard palladium (Pd) solution, specifically the Plasma CAL ICP/ICPMS Standard‐Palladium 1000 μg/mL preserved in 10% HCl (SCP Science, Canada). This standard solution was diluted with reconstituted water, which was used for culturing *D. magna* , to prepare the exposure solution. The water temperature was consistently regulated at 20°C ± 2°C under a 16‐h light/8‐h dark cycle.

Water samples were analyzed to verify the actual concentrations in the exposure solutions. For acute tests, samples were collected at the start and end of the experiment. During chronic tests, water was renewed every 2 days to maintain stable exposure conditions. Water measurements were taken every other water change, both before and after. Prechange sampling assessed the concentration in the solution that remained with the organisms for 2 days, whereas postchange sampling measured the concentration in the newly refreshed solution. To eliminate algae in prechange sampling, water was filtered through a 0.45‐μm membrane (Millipore) using a syringe. The filtered samples were acidified with 1% HNO_3_ and 5% HCl (v/v) and analyzed by inductively coupled plasma triple quadrupole mass spectrometry (ICP‐MS/MS, 8900, Agilent Technologies; Agilent 2022) at Université de Montréal. Further details on Pd analysis quality control via ICP‐MS/MS are provided in the Supporting Information S1.

### 2.3 Acute Toxicity Tests

The acute toxicity test involved exposing newborn *D. magna* (≤ 24 h old) to a series of nominal Pd concentrations ranging from 2 to 180 μg/L, alongside a control, and monitoring mortality. Four replicates were performed for each concentration, with each replicate consisting of five‐to‐eight daphnids maintained in a borosilicate glass tube containing 2 mL of test solution per individual (i.e., 10–16 mL per tube) (OECD 2004). Three preliminary range‐finding assays were conducted to define an appropriate concentration gradient of Pd for acute toxicity assessment. Subsequently, the definitive 48‐h acute toxicity test was performed in duplicate, with each test comprising four independent replicates per concentration. Exposures were conducted under a static system without the addition of food, in accordance with the CEAEQ and OECD guideline 202 protocols (OECD 2004; CEAEQ 2021). Parameters such as dissolved oxygen, hardness, and pH were recorded at the beginning and end of the exposure period, and no significant changes were observed.

The aim of the experiment was to determine the Pd concentrations leading to 10% (LC_10_) and 50% (LC_50_) mortality in *D. magna* following 48 h of exposure, under conditions devoid of natural organic matter. Mortality was determined by the absence of appendage and antenna movement, and the cessation of heartbeats, observed under a binocular microscope. Preliminary trials were conducted to define an appropriate concentration range for the acute toxicity tests.

The reference test with a standard toxicant for toxicity assessments (i.e., positive control) following OECD and CEAEQ protocols was conducted using potassium dichromate (K_2_Cr_2_O_7_). This test aimed to assess the sensitivity of the test organisms and ensure that experimental conditions were suitable for detecting potential toxicity.

Furthermore, the thermodynamic simulation software Visual MINTEQ 3.1 was employed to estimate the chemical speciation of Pd in the absence of DOC. This analysis enabled the identification of the species present in the exposure environment, incorporating key input parameters such as the Pd concentration, inorganic ligands (e.g., Cl^−^, Ca^2+^, and SO_4_^2−^), and essential physicochemical factors, including pH and temperature, which are critical in determining metal speciation. However, Visual MINTEQ 3.1 did not contain the thermodynamic data required to model the Pd (OH)_2(aq)_ species. To address this limitation, the relevant formation constants (log β) and reaction enthalpies (ΔrH^0^) for this species were manually incorporated, based on the data provided by Duro et al. (2006): Pd^2+^ + 2H_2_O ↔ Pd (OH)_2_(aq) + 2H^+^: log *β* = −3.79; ΔrH^0^ = 15.29 kJ/mol.

In the presence of organic matter, the thermodynamic software Windermere Humic Aqueous Model (WHAM7) was used to predict the chemical speciation of Pd in exposure solutions. This approach enabled a detailed evaluation of Pd distribution across free ionic forms, inorganic complexes, and organic complexes. Measured values of key input parameters—including pH, temperature, and the concentrations of Pd, inorganic species, and organic matter components—were used to ensure an accurate representation of the experimental conditions. Organic complexation was modeled by incorporating specific concentrations of fulvic acid (FA), considering that DOM contains 50% carbon and 65% of DOM is active in complexing Pd (active FA = DOC (mg/L) × 2 × 0.65) (Bryan et al. 2002).

#### 2.3.1 Effect of DOC on Mortality and Bioaccumulation

The DOC used in this study originated from a reference sample of natural organic matter (NOM) collected from the Suwannee River (NOM, Catalog Number 1R101N, United States). The sample was processed through reverse osmosis and cation exchange to remove inorganic components. The NOM was supplied as a 400 mg/L stock solution, which was diluted with deionized water, equilibrated, and sonicated to ensure complete dissolution and uniform dispersion.

An acute toxicity test was conducted to assess the effect of DOC on Pd‐induced mortality in *D. magna* neonates, used to determine the 48‐h LC_50_. DOC concentrations of 0.8‐, 1‐, 2‐, 5‐, and 8‐mg C/L were tested in conjunction with nominal Pd concentrations of 0, 60, 80, 100, 120, 140, 160, and 180 μg/L. At the beginning and end of the 48‐h exposure period, concentrations of Pd and DOC in the water were monitored. DOC levels were determined using a DC‐190 total organic carbon (TOC) analyzer, whereas Pd concentrations were quantified using ICP‐MS/MS.

Adult *D. magna* , visually identified based on size, were used for bioaccumulation tests to ensure sufficient dry biomass (~1 mg) for accurate Pd quantification by ICP‐MS, as neonates did not provide enough mass for reliable measurements. Organisms were exposed for 48 h in glass Pyrex beakers containing 200 mL of test solution with a nominal Pd concentration of 40 μg/L and varying DOC concentrations (0.57, 0.65, 1.0, and 1.4 mg C/L), with 10 individuals per beaker and three to six replicates per DOC concentration. The Pd concentration (40 μg/L) was chosen based on prior acute toxicity tests, where it caused sublethal effects without significant mortality. The DOC range (0.57–1.4 mg C/L) was also based on earlier tests, in which environmentally relevant concentrations (1–8 mg C/L) did not cause mortality. Slight mortality was only observed at 0.8 mg C/L when combined with the highest Pd concentrations, prompting the use of a lower DOC range to investigate potential effects on Pd bioaccumulation. At the end of the exposure, daphnids were rinsed by allowing them to swim in Milli‐Q water for approximately 5 min to remove any Pd adsorbed on their exoskeletons. They were then transferred into 1.5‐mL centrifuge tubes, freeze‐dried (lyophilized), and weighed to determine their dry mass.

The samples were then digested in Teflon vessels using 600 μL of concentrated HNO_3_ (trace metal grade, Fisher Scientific, purified by subboiling with the Savillex DST‐1000 purification system) and 200 μL of HCl (trace‐metal grade, Fisher Scientific) in a pressure cooker (15 psi) at 120°C (All American, model 50X‐120 V) for 3 h. To ensure complete oxidation of organic material, 250 μL of hydrogen peroxide (30% H_2_O_2_, OPTIMA grade, Fisher Scientific) was added to the sample and left overnight. The digested solutions were transferred to 15‐mL trace‐metal‐free tubes (VWR) and diluted with Milli‐Q water to a final volume of 15 mL. Pd bioaccumulation was quantified using ICP‐MS/MS. Details on quality control procedures for this analysis are provided in Supporting Information S1.

### 2.4 Chronic Toxicity Tests

Neonate daphnids less than 24 h old were exposed to various nominal Pd concentrations: 0, 5, 12, 30, 48, 75, and 120 μg/L to assess the impact of Pd on the reproductive rate of the daphnids. Each concentration was tested with 10 replicates, using glass beakers containing 60 mL of exposure solution, each holding a single daphnid. Prior to this definitive 15‐day chronic toxicity test, a preliminary test was conducted to validate the selected Pd concentrations and experimental conditions, including food quantity and frequency. The temperature was maintained at 20°C ± 2°C with a 16‐h light/8‐h dark cycle in an incubator. During the exposure period, as well as at the beginning and end, parameters including dissolved oxygen, hardness, and pH were measured, and no significant changes were observed. Although OECD guideline 211 protocol (OECD 2012) prescribes a 21‐day test period, this study reduced the exposure to 15 days to avoid further parental mortality that has already been observed during the initial days, especially for the highest concentrations, enabling further sublethal testing.

This semistatic test involved daily feeding of the daphnids with an algal solution (*Raphidocelis subcapitata*), adjusted according to the organic carbon intake required per organism. The carbon content of the algal solution used was 180 mg C/L. From Days 1 to 6, neonates were fed 0.5 mL per day, corresponding to a carbon intake of 0.1 mg C/L. Once they matured to adults, from Days 6 to 15, the feeding volume was increased to 1 mL per day, equating to a carbon intake of 0.2 mg C/L. Additionally, a yeast‐cerophyll‐trout chow (YCT) mixture was supplemented at 0.5 μL/mL during each water renewal. The number of live offspring produced by surviving parents was recorded daily for each replicate to evaluate reproduction. The toxic effect of Pd on reproduction was determined by identifying the concentration that resulted in a 50% reduction in reproduction in *D. magna* . Additionally, at the end of the exposure period, analyses of respirometry, growth, parent survival, and bioaccumulation were conducted.

#### 2.4.1 Respirometry

At the end of the reproduction trial, the respiration rate of live parental *D. magna* was assessed using a glass microplate system (Loligo system, SY25210, Viborg, Denmark) to compare Pd‐exposed organisms with the control group. The number of surviving daphnids used in the analysis varied based on nominal Pd concentrations: 10 individuals for 0 μg/L, 8 for 5 and 12 μg/L, 7 for 30 μg/L, and 2 for 48 μg/L. This system consists of four microplates, each containing 24 wells of 200 μL with serial oxygen sensors, and each well containing a single daphnid. The oxygen consumption per well was measured using the MicroResp software (MicroResp, Version 1) when air saturation was between 80% and 60%. This range was selected to ensure more accurate results, as daphnids reduce their oxygen consumption when oxygen availability decreases (Yashchenko et al. 2016). As a control solution, blanks consisting only of the daphnid culture water were used, corresponding to three wells per microplate.

#### 2.4.2 Bioaccumulation

Bioaccumulation was assessed in both acute and chronic toxicity tests. Unlike the acute exposure experiment described in Section 2.3.1, where Pd bioaccumulation was evaluated in the presence of varying DOC concentrations, the present assessment was conducted without DOC to isolate the direct effects of Pd. In the acute test, adult *D. magna* were exposed for 48 h to nominal Pd concentrations of 0, 2, 5, 80, 100, 150, and 200 μg/L. In the chronic test, Pd bioaccumulation was evaluated at the end of the 15‐day exposure period, following the completion of reproduction and respirometry assessments.

For both tests, Pd accumulation in *D. magna* was quantified using the same digestion and analytical procedures described in Section 2.3.1, with slight modifications in the chronic test to ensure complete digestion and accurate Pd detection given the lower biomass (0.1–0.5 mg dry weight). Specifically, organisms were digested in 200 μL of HNO_3_, 100 μL of HCl, and 80 μL of hydrogen peroxide, resulting in final acid concentrations of 4% and 1%. Pd concentrations were then determined by ICP‐MS/MS under identical quality control conditions.

### 2.5 Data Analysis

Statistical analyses were performed using RStudio (R Core Team 2024), with data visualization carried out using the ggplot2 package. The significance threshold was established at *α* = 0.05. Normality was assessed using the Shapiro–Wilk test, with a *p* value > 0.05, and homogeneity of residuals was verified using the Brown–Forsythe test.

Dose–response relationships for reproduction and survival were modeled using nonlinear regression with log‐logistic models from the *drc* package (Ritz et al. 2015). A four‐parameter log‐logistic model (LL.4) was used to analyze the total number of offspring per *Daphnia magna* , whereas three‐parameter log‐logistic models (LL.3) were applied for the number of broods and parental survival. These models were selected based on the biological characteristics of the endpoints and model convergence. Effective concentrations (EC_10_ and EC_50_) were derived from these models when applicable. For Pd bioaccumulation and respiratory results, linear regression models were performed using the “lm” function, reporting the *R*‐squared (*R* ^2^) value and regression line for models meeting the significance threshold (*α* = 0.05). Dry weight responses across treatments were compared using one‐way analysis of variance (ANOVA), followed by Tukey's post hoc test for group mean comparisons. All data are presented as mean ± standard error of the mean (SEM).

## 3 Results

### 3.1 Palladium Concentrations in Exposure Solutions and Test Validation

**FIGURE 1:**
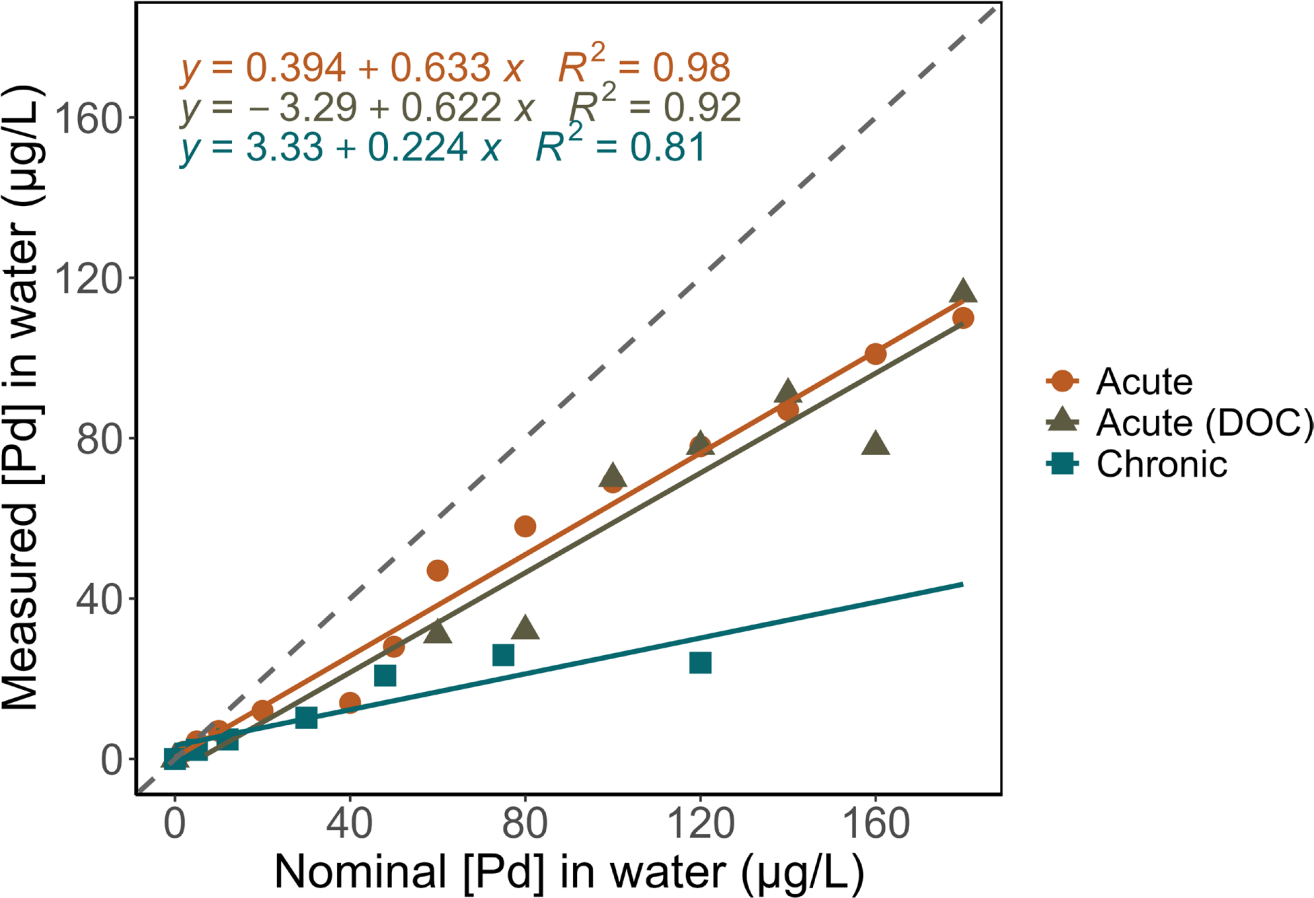
Measured Pd concentrations as a function of nominal Pd concentrations (μg/L) in acute and chronic tests. For acute tests, data represent the mean of two measurements per concentration, taken before and after 48 h of exposure (*n* = 2 per concentration). For chronic tests, data represent the mean of two averaged values per concentration: one from three prerenewal measurements and one from three postrenewal measurements, with this procedure repeated three times over a 15‐day period (*n* = 3). For Pd concentrations measured in the presence of DOC, only the final measurement after 48 h of exposure is available. Linear models are statistically significant (*p* < 0.05).

The determination of dissolved Pd concentrations in exposure solutions from both acute and chronic tests confirmed that no Pd was detected in the control samples (detection limit of 0.1 ng/L; see Section S1 for quality control details). However, in the amended solutions, Pd concentrations deviated from nominal values after 48 h of exposure (Figure 1). In the acute test without DOC, lower nominal concentrations such as 2 and 5 μg/L showed relatively high recovery rates of 90% and 88%, respectively. As nominal concentrations increased, a general trend of decreasing recovery was observed, with a notable drop to 35% at 40 μg/L and 56% at 50 μg/L. This decline in recovery was particularly evident at concentrations above 60 μg/L, where recovery ratios consistently remained below 80%. In contrast, for the acute test with DOC, recovery patterns were less predictable. For example, at 60 and 80 μg/L, recoveries were 52% and 40%, respectively, whereas at higher concentrations, such as 100 and 120 μg/L, recoveries improved to 70% and 65%. In the chronic test, recovery rates were generally low and variable, particularly at higher concentrations. For example, at 120 μg/L, the lowest recovery of 20% was recorded, and at lower concentrations, recoveries ranged from 33% at 30 μg/L to 42% at both 12 and 48 μg/L.

All results are reported using measured concentrations to ensure accurate representation of organism exposure levels. For acute tests, exposure concentrations were determined as the mean of initial and final measurements. For the chronic 15‐day exposure, dissolved Pd concentrations were measured at the beginning and end of the following exposure intervals: Days 0–2, 6–8, and 12–14. These time points were selected to provide representative measurements across the entire experimental period. For each exposure concentration, a mean was first calculated from the three prerenewal measurements and another from the three postrenewal measurements. A final mean exposure concentration was then derived as the average of these two means.

The reference tests conducted with potassium dichromate (K_2_Cr_2_O_7_) determined 24‐h LC_50_ values of 0.7 and 0.8 mg/L, which fall within the range of 0.6–2.1 mg/L recommended by OECD (2012). Thermodynamic analysis using the Visual MINTEQ 3.1 simulation software estimated the chemical speciation in the exposure solution. The results indicated that at neutral pH and in the absence of natural organic matter, the predominant species was PdOH_2(aq)_, accounting for > 99.9% of the Pd in solution, all other species being < 0.1%.

The thermodynamic software WHAM was employed to predict Pd speciation in exposure solutions containing DOC. Speciation was calculated for Pd concentrations ranging from 31 to 116 μg/L across DOC levels of 0.8, 1.0, and 1.4 mg/L. The results indicated that, at a constant Pd concentration, the proportion of Pd bound to FA increased with DOC concentration. However, as the Pd concentration increased, the proportion of Pd bound to FA decreased, particularly at lower DOC concentrations. For example, at 0.8 mg/L DOC, the FA‐bound fraction decreased by 32% (from 98% to 66% between 31 and 116 μg/L Pd) (Figure S1).

During the acute and chronic toxicity experiments, water quality measurements adhered to the ranges specified by the OECD guidelines. A detailed table containing the pH values for each test is available in the Supporting Information (Table S1). Water hardness, measured as the sum of calcium and magnesium concentrations, fell within the prescribed range of 150–170 mg/L. Additionally, dissolved oxygen concentrations were consistently maintained above 6 mg/L throughout the experiments.

### 3.2 Acute Toxicity Test With and Without DOC

**FIGURE 2:**
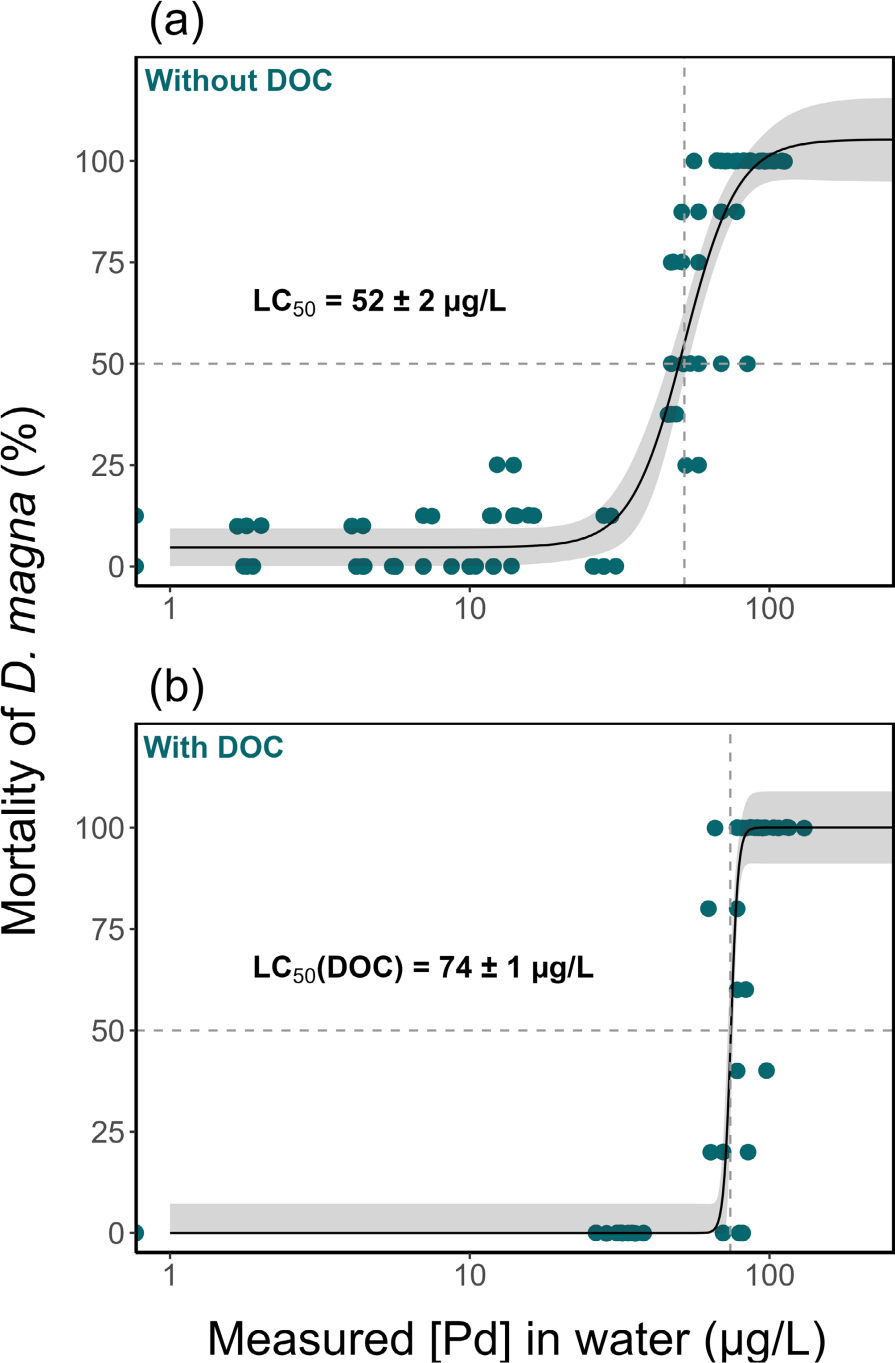
Concentration‐response curves demonstrating the effect of Pd on the mortality of *D. magna* over a 48‐h exposure period in the presence (a) and absence (b) of dissolved organic carbon (DOC) (*n* = 4). Each dot represents the percentage of mortality within a replicate. The pH ranged from 7.3 to 8.1 with DOC and from 6.6 to 7.6 without DOC, varying from low to high concentrations. In the presence of DOC (b), the measured DOC concentration was 0.8 mg C/L. The medium used was *Daphnia* culture water without M4 medium. Shaded areas represent the 95% confidence interval. See Figure S4 for same figure with nominal concentrations.

The effects of Pd were observed in exposed *D. magna* , showing an increase in mortality with increasing measured exposure concentrations, reaching 100% at the highest concentration tested. The concentration‐response curve (Figure 2a) also allowed for the determination of a 48‐h LC_50_ and LC_10_, with respective values of 52 ± 2 and 33 ± 3 μg/L.

Additional experiments were conducted with DOC into the exposure solution to evaluate its effect on the acute mortality of *D. magna* across a gradient of measured Pd concentrations. No mortality was observed at DOC concentrations of 1, 2, 5, and 8 mg/L. However, at the lowest DOC level of 0.8 mg C/L, 100% mortality occurred at Pd measured concentrations of 78 μg/L and higher. This resulted in a concentration–response curve (Figure 2b), yielding an LC_50_ of 74 ± 1 μg/L and an LC_10_ of 70 ± 2 μg/L.

### 3.3 Chronic Toxicity of Palladium

#### 3.3.1 Reproduction

The impact of Pd on *D. magna* reproduction was assessed following a 15‐day chronic exposure. Various reproductive and physiological parameters were measured, including the total number of live offspring per *D. magna* per day, the total number of broods per *D. magna* per day, the timing of the first brood, and the weight and survival of adult *D. magna* . An increase in parental mortality, exhibiting a clear concentration–response relationship, was observed during the study, leading to the premature termination of the experiment at Day 15. In accordance with OECD Guideline 211, such mortality is considered a treatment‐related effect. Consequently, replicates in which adult organisms produced offspring before subsequently dying are retained in the analysis, as the observed mortality is concentration dependent and thus attributable to the test substance.

**FIGURE 3:**
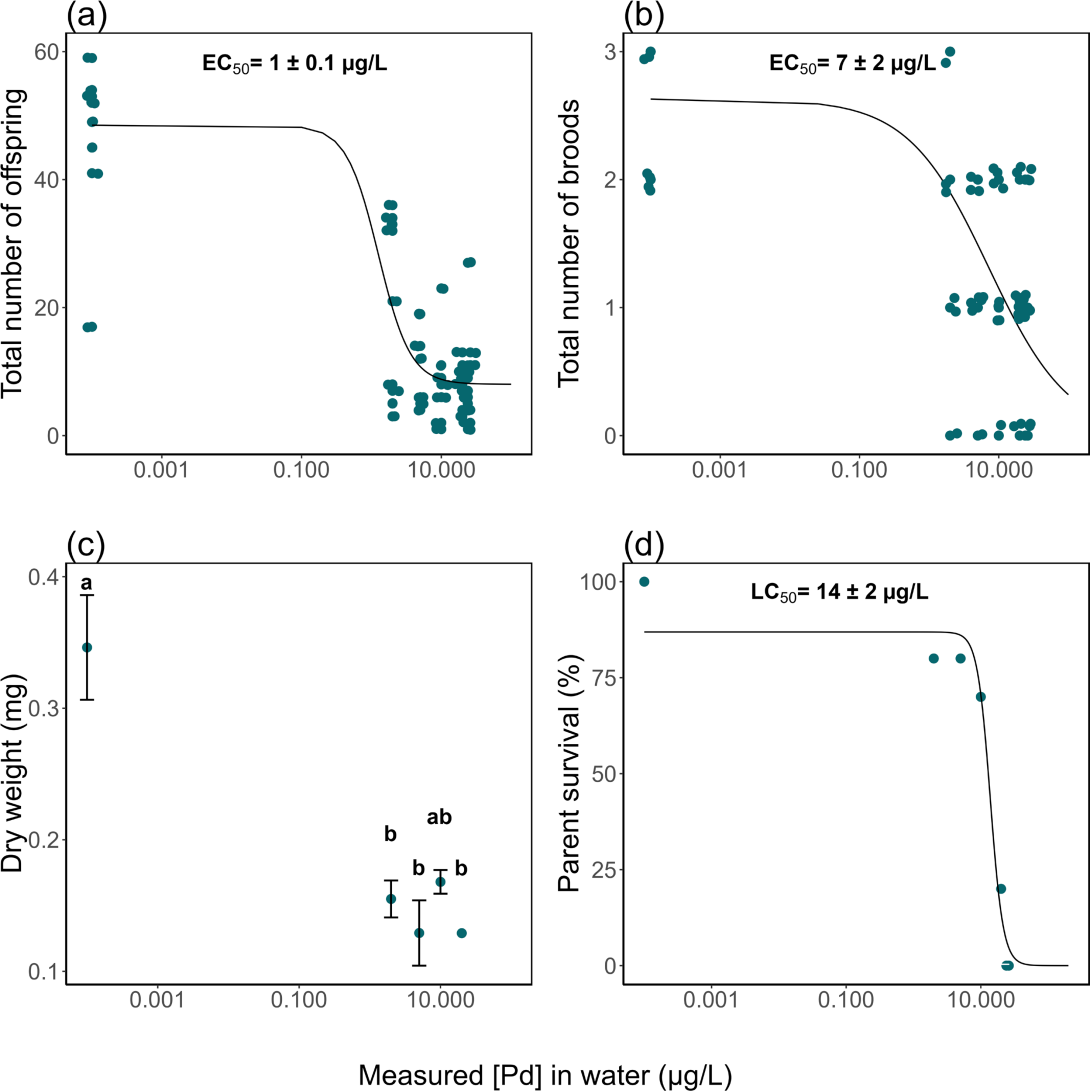
(a) Total number of offspring per *D. magna* (*n* = 10 for 0 μg/L; *n* = 9 for 2 and 5 μg/L; *n* = 8 for 10 μg/L; *n* = 7 for 20 μg/L; *n* = 8 for 24 μg/L; and *n* = 5 for 26 μg/L). (b) Total number of broods per *D. magna* (*n* = 10). (c) Dry weight of the parent *D. magna* (mg) (*n* = 10 for 0 μg/L; *n* = 7 for 2 μg/L; *n* = 8 for 5 μg/L; *n* = 2 for 10 μg/L; and *n* = 1 for 20 μg/L). Significant differences (*p* < 0.05) are indicated by distinct letters. (d) Survival of the parent *D. magna* (*n* = 10) as a function of measured Pd concentrations in water (measured [Pd] in water, μg/L). The values represent mean ± standard error. Nonlinear regressions were fitted using the drm function in the drc package (R): a four‐parameter log‐logistic model (LL.4) for panel (a) and three‐parameter log‐logistic models (LL.3) for panels (b) and (d) (all models significant at *p* < 0.05). See Figure S5 for same figure with nominal concentrations.

The total number of live offspring (Figure 3a) significantly decreased with increasing Pd concentrations compared to the control, yielding an EC_50_ of 1 ± 0.1 μg/L. A similar pattern was observed in the total number of broods (Figure 3b), where a significant reduction was detected across all exposure concentrations, resulting in an EC_50_ of 7 ± 2 μg/L. However, the timing of the first brood did not show significant differences across treatments (Figure S2). The dry weight of the parent *D. magna* (Figure 3c) exhibited a reduction compared to the control, with the EC_50_ estimated to be ≥ 5 μg/L. This threshold was chosen because no significant difference was observed between 2 and 5 μg/L, indicating that the 50% effect level is unlikely to be below 5 μg/L. Parental survival (Figure 3d) also significantly declined, reaching 100% mortality at the two highest measured concentrations (24 and 26 μg/L), with an LC_50_ of 14 ± 2 μg/L.

#### 3.3.2 Respirometry

**FIGURE 4:**
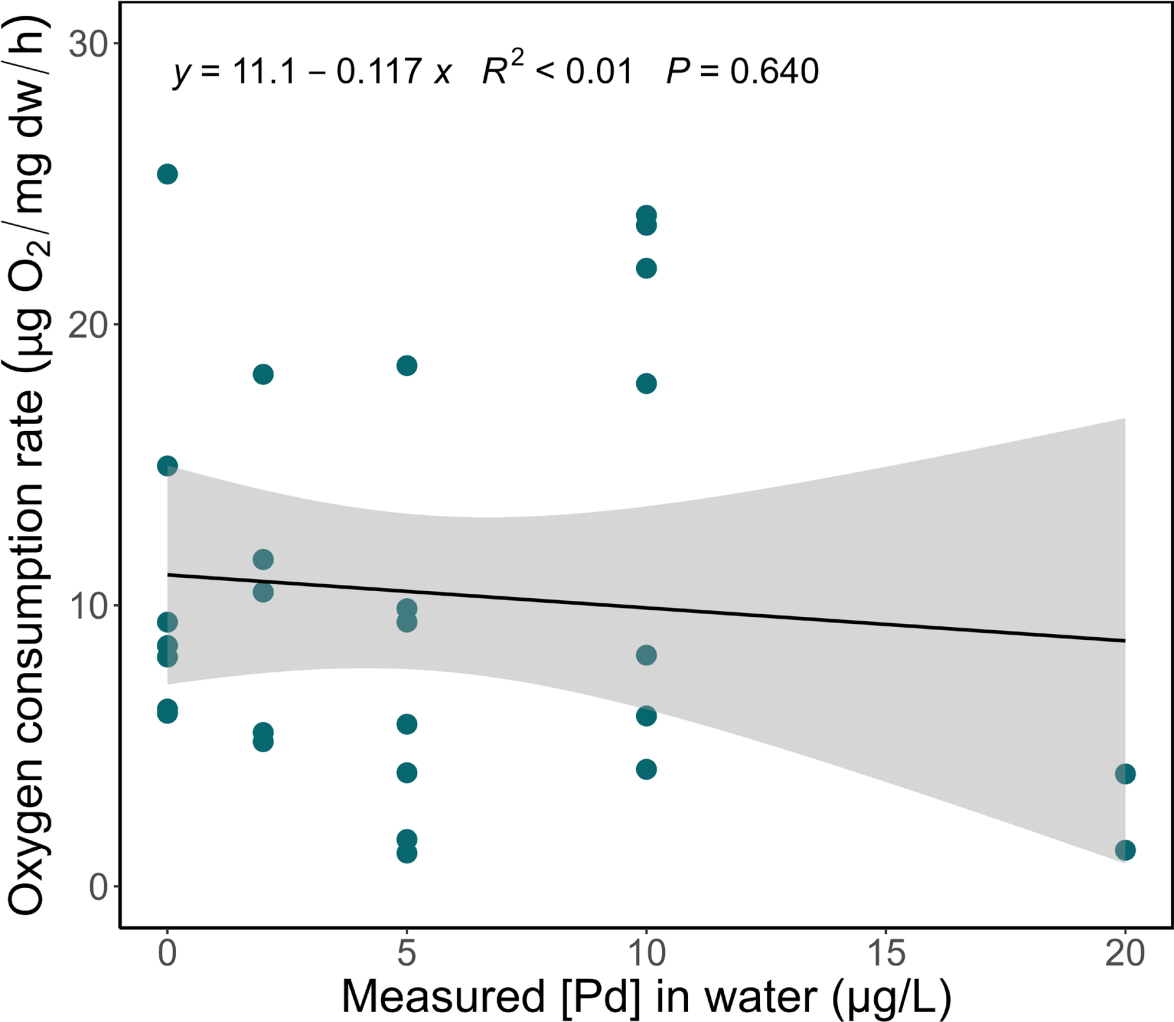
Oxygen consumption rate in adult *D. magna* after 15 days of exposure as a function of measured Pd concentrations in water, standardized per mg dw of daphnid biomass (μg O_2_/mg dw/h) (*n* = 7 for 0 μg/L; *n* = 6 for 2 μg/L; *n* = 7 for 5 and 10 μg/L; and *n* = 2 for 20 μg/L). Linear model is significant (*p* < 0.05). See Figure S6 for same figure with nominal concentrations.

Oxygen consumption rates decreased significantly across the tested Pd concentrations (Figure S3). At the highest exposure (20 μg/L), the oxygen consumption rate was the lowest and reached 0.08 μg O_2_/h. However, these results are considered an artifact of the weight loss as exposure rises. Thus, smaller individuals (highest exposure) were consuming less oxygen per hour. However, after normalizing these results based on the dry weight of *D. magna* , no significant differences were recorded compared to the controls (Figure 4). Indeed, the daphnids were smaller at higher exposure concentrations (Figure 3c), thus reducing the oxygen consumption rate in the microplate wells.

### 3.4 Bioaccumulation of Pd

**FIGURE 5:**
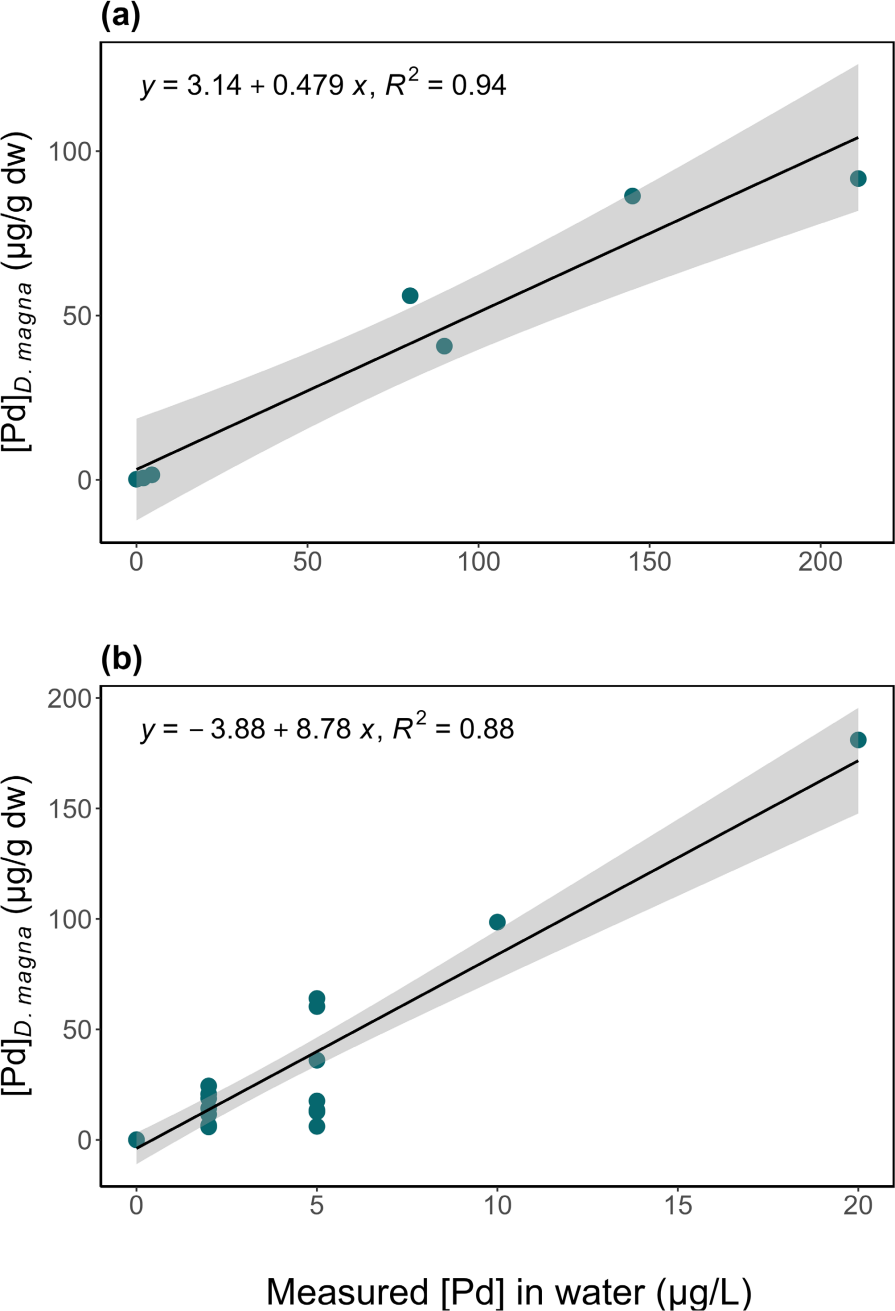
Palladium bioaccumulation ([Pd], μg/g dw) in *D. magna* as a function of measured aqueous Pd concentrations (measured [Pd] in water μg/L). Panel (a): acute exposure (48 h); each dot represents a pooled sample of 10 individuals per concentration. Panel (b): chronic exposure (15 days); each dot represents an individual surviving adult. Linear models are significant (*p* < 0.05). See Figure S7 for same figure with nominal concentrations.

The bioaccumulation of Pd was assessed in adult *D. magna* at the end of both acute and chronic toxicity tests. The Pd bioaccumulation presented a strong linear correlation with measured aqueous Pd exposure (Figure 5). Note that Pd bioaccumulation was similar after 2 days at high concentrations and after 15 days at low concentrations.

#### 3.4.1 Influence of DOC on Palladium Bioaccumulation

**FIGURE 6:**
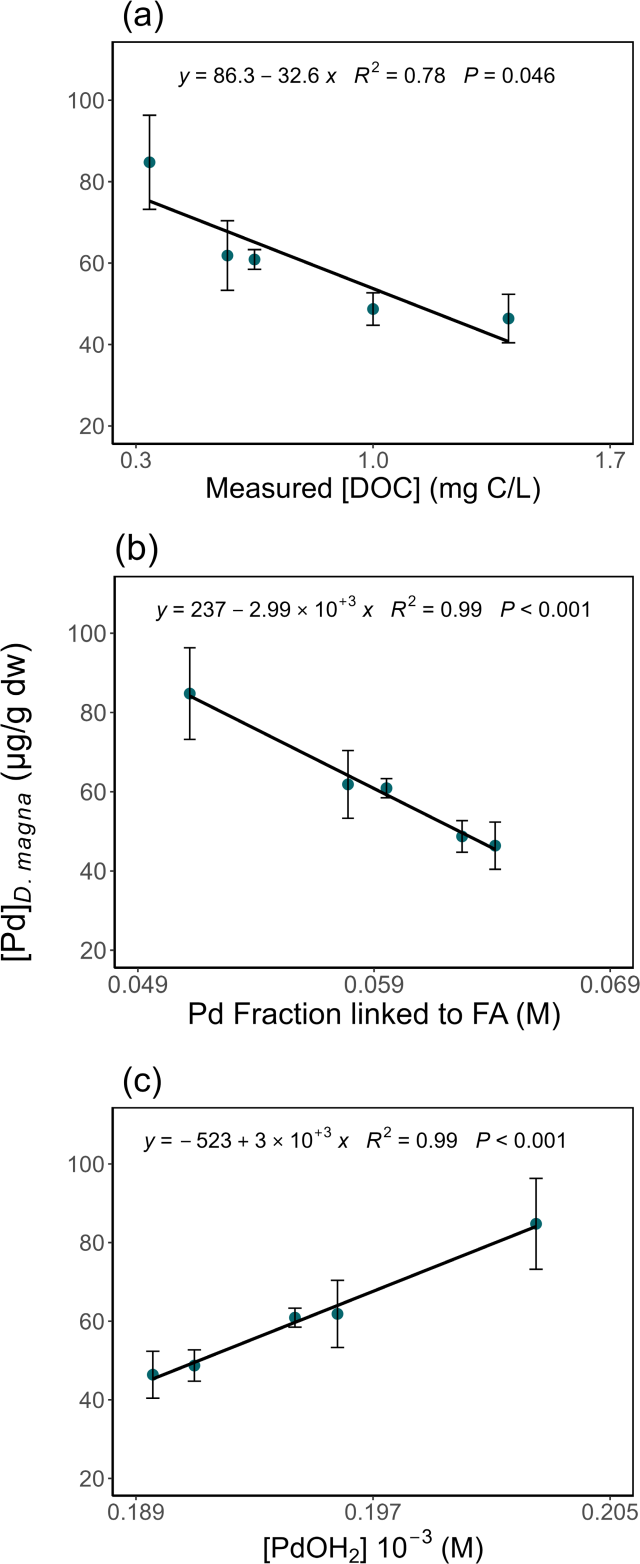
Pd concentrations in *D. magna* ([Pd] _*D. magna*_ , μg/g dw) after 48 h exposure (*n* = 3 for 0.57 and 0.65 μg/L and *n* = 6 for 0.34, 1, and 1.4 μg/L per concentration): (a) as a function of dissolved organic carbon concentrations (measured [DOC], mg C/L); (b) as a function of Pd fraction linked to fulvic acid (FA, M); and (c) as a function of PdOH_2_ ([PdOH_2_] 10^−3^, M). Pd exposure concentration used in this experiment was 27 μg/L (measured). Linear models are significant (*p* < 0.05).

DOC played a crucial role in modulating Pd bioaccumulation. After 48 h of exposure to a measured Pd concentration of 27 μg/L, linear regression analysis revealed a strong inverse relationship between bioaccumulation and DOC concentrations (Figure 6a). As DOC levels increased (0.57, 0.65, 1.0, and 1.4 mg C/L), Pd bioaccumulation decreased by approximately 30%–40%, reaching up to a 50% reduction at the highest DOC level, compared to the control (0.34 mg C/L) where no DOC was added. Consistently, WHAM simulations, which considered DOC as fulvic acids (FA), revealed an inverse relationship between Pd bioaccumulation and its fraction complexed with FA (Figure 6b). As this fraction increased, Pd accumulation in *D. magna* significantly decreased, suggesting that complexation with FA reduces Pd bioavailability. Conversely, the estimated fraction considered as PdOH_2_ exhibited a positive correlation with bioaccumulation (Figure 6c). Higher PdOH_2_ concentrations were associated with increased Pd uptake, indicating that this species is likely the most bioavailable. To further evaluate the bioavailability of Pd species, uptake rate constants (*k*_*uw*_) were determined for PdOH_2_ and Pd^2+^. The uptake rate constant for PdOH_2_, estimated from the slope of the uptake rate (nmol/g/day) as a function of aqueous PdOH_2_ concentration (nmol/L), was 0.014 L/g/day. This value is consistent with reported *k*_*uw*_ values for *D. magna* and is comparable to elements with low aqueous uptake, such as selenium (*k*_*uw*_ = 0.18 L/g/day) (Tsui and Wang 2007). However, it is possible that this value underestimates the actual uptake rate constant, as Pd losses over the 48‐h exposure were not accounted for. Conversely, the uptake rate constant calculated for free Pd^2+^ (*k*_*uw*_ = 2 × 10^8^ L/g/d) is unrealistically high, further supporting the hypothesis that PdOH_2_ is the predominant bioavailable species. Despite these findings, our experimental design does not allow for a definitive identification of the internalized Pd species.

To enhance the clarity and accessibility of the findings, a summary table is presented below (Table 1), consolidating the main effect concentrations (LC_10_, LC_50_, and EC_50_) derived from both acute and chronic exposure. Reported values include the corresponding 95% confidence intervals where applicable. This table encompasses endpoints related to survival, reproduction, and growth, as well as bioaccumulation data modeled using linear regression.

**TABLE 1:** Summary of effect concentrations and bioaccumulation parameters for *D. magna* exposed to palladium.

Exposure type	Endpoint	Effect concentration (μg Pd/L, measured)	95% CI (μg/L)
Acute (48 h)	LC_10_ (mortality) LC_50_ (mortality)	33 52	27–39 47–57
Acute (48 h + DOC)	LC_10_ (mortality) LC_50_ (mortality)	70 74	67–73 73–76
Chronic (15 days)	EC_50_ (reproduction, offspring/female)	1.3	1.1–1.5
	EC_50_ (reproduction, number of broods)	7	2–12
	EC_50_ (dry body weight)	≥ 5	na
	LC_50_ (survival)	14	9–19
Acute (48 h)	Linear model (bioaccumulation)	*y* = 3.14 + 0.479 × *x* *R* ^2^ = 0.94	na
Chronic (15 days)	Linear model (bioaccumulation)	*y* = −3.88 + 8.78 × *x* *R* ^2^ = 0.88	na

## 4 Discussion

### 4.1 Importance of Measuring Aqueous Pd Exposure

The concentrations of Pd in exposure solutions decreased significantly compared to nominal values, with the greatest reductions observed at higher concentrations (Figure 1). This pattern strongly indicates precipitation as the dominant mechanism for Pd loss, as elevated metal concentrations typically enhance this process. As a class B metal, Pd readily forms stable covalent bonds with soft ligands and functional groups such as sulfur, phosphorus, and hydroxyl groups (‐OH) (Duffus 2002), which may further contribute to its removal from solution. Adsorption onto hydroxyl groups on the inner surfaces of glass vials may account for part of the observed losses. Moreover, the 24‐h preparation and equilibration period for the exposure solutions, intended to ensure stability, likely allowed substantial Pd loss prior to the start of the experiments. During chronic exposures, Pd recovery became increasingly variable, a trend attributable to several biotic and abiotic factors. Daily feeding with algae (*Raphidocelis subcapitata*) and YCT (yeast, cerophyll, and trout chow) may have influenced Pd speciation and reduced bioavailability due to the presence of organic ligands capable of binding metals. Notably, YCT filtrate has been shown to markedly alter metal toxicity: Kolts et al. (2008) reported a 27‐fold increase in the 48‐h EC_50_ for silver in *Caenorhabditis elegans* in the presence of YCT filtrate compared to natural DOM, whereas Hylton and Tsui (2021) demonstrated that YCT reduced acute mercury toxicity in *D. magna* , likely through complexation of dissolved metal species. These findings underscore the potential of YCT to modulate Pd bioavailability during testing. Residual algae retained on filtration membranes may have further promoted Pd loss through passive adsorption. In parallel, the production of offspring during the experiment may have affected Pd concentrations (via adsorption and excretion of metabolites), contributing to the variability in recovery observed. These factors, combined with the inherent chemical properties of Pd, may cause measured concentrations in the exposure solutions to consistently fall below their nominal counterparts. When nominal concentrations are used for interpretation, this discrepancy can lead to an overestimation of the actual exposure levels experienced by organisms. Therefore, accurate measurement of Pd concentrations is essential in studies involving this metal.

### 4.2 Acute Pd Toxicity

During a 48‐h acute exposure, Pd resulted in significant mortality in *D. magna* , with an LC_50_ of 52 ± 2 μg/L and an LC_10_ of 33 ± 3 μg/L (Figure 2). In contrast, Zimmermann et al. (2017) reported a 48‐h LC_50_ of 14 μg/L based on measured concentrations. The discrepancy between the LC_50_ values observed in our study and those reported by Zimmermann et al. (2017) may stem from differences in biological and physicochemical conditions. Although both studies employed a standardized reference toxicant as a positive control, the origin of *Daphnia* used could influence sensitivity to Pd. Our study utilized laboratory‐cultured organisms, whereas Zimmermann et al. (2017) used dormant eggs from the DaphToxKit, which may exhibit variations in sensitivity and acclimatization. Moreover, water chemistry, particularly parameters such as hardness, is a well‐documented factor affecting metal toxicity (Borgmann et al. 2005; Adams et al. 2020). Variability in these parameters between studies could influence the bioavailability and toxicity of Pd, potentially explaining the observed differences in LC_50_ values.

Other studies have documented a 48‐h (nominal) LC_50_ of 142 μg/L for *Tubifex tubifex* and a 96‐h (nominal) LC_50_ of 293 μg/L for zebrafish ( *Danio rerio* ) (Khangarot 1991; Chen et al. 2015). These LC_50_ values are substantially higher than environmental concentrations of Pd, which are typically in the nanogram per liter range (Hu et al. 2005; Liu et al. 2021). These findings highlight pronounced species‐specific differences in Pd sensitivity, with *T. tubifex* exhibiting significantly greater susceptibility compared to *D. rerio* . Similarly, Khangarot and Das (2009) demonstrated that sensitivity varies notably across species, with *D. magna* being more sensitive than *Cypris subglobosa* . This variability underscores the need to evaluate multiple species in toxicological assessments to fully understand the environmental risks associated with metal exposure.

In this study, Pd exhibited moderate toxicity, with an LC_50_ of 52 μg/L, positioning it between highly toxic metals such as Hg and Ag and less toxic elements like Ni and Fe (Khangarot and Das 2009). Quantitative Ion Character Activity Relationship (QICAR) modeling for *D. magna* further supports this classification, indicating that Pd (IV) and Pd (II) exhibit significant toxicity, with EC_50_ values of 3 and 18 μg/L, respectively (le Faucheur et al. 2021). These results position Pd among the more hazardous elements, surpassing the toxicity of Cu and Cr and approaching that of Ag. This classification aligns with findings from Khangarot (1991), who reported Pd to be less toxic for an oligocheate than Ag, Se, and Cd but more toxic than Cu and Cr. Notably, the mode of action of Pd differs from that of more acutely toxic metals. Although elements such as Ag, As, and Cd primarily induce direct physiological toxicity, Pd may also be associated with physical immobilization in *D. magna* (Okamoto et al. 2015), by adhering to its body and hindering movement and feeding.

The establishment of these effect concentrations is crucial for a complete assessment of the risks associated with this metal. The current literature on the toxicity of Pd is not only limited in scope but also hindered by several critical experimental deficiencies. Specifically, many studies lack robust methodologies and fail to report essential parameters such as measured exposure concentrations, the extent of metal bioaccumulation, and detailed chemical speciation. These omissions significantly undermine the reliability of toxicity assessments, as they are crucial for understanding the actual exposure levels, the biological availability of the metal, and its potential interactions within environmental and biological systems (Batley and Campbell 2022). Additionally, the impact of natural organic matter on Pd toxicity is often overlooked, despite its potential to significantly affect metal solubility, transport, and uptake in freshwater environments (Adams et al. 2020). This highlights the need for further investigation into the role of Pd complexation in modulating its toxic effects.

### 4.3 Influence of DOC on Pd Bioaccumulation, Toxicity, and Speciation

This study highlights the pivotal role of DOC in modulating the bioavailability and toxicity of Pd to *D. magna* . Elevated DOC concentrations significantly reduced Pd bioaccumulation (Figure 6), consistent with the Biotic Ligand Model (BLM), which predicts a decline in metal bioavailability due to enhanced complexation with dissolved organic matter. However, the complexity of Pd–DOC interactions is underscored by previous studies reporting divergent effects. For instance, Sures and Zimmermann (2007) observed a decrease in Pd uptake by *D. polymorpha* when exposed to humic water, whereas Zimmermann et al. (2002) reported increased Pd uptake under similar humic conditions for the same species. A similar variability has been observed for platinum (Pt): Hourtané et al. (2022) found that humic substances at concentrations of 10 and 20 mg C/L enhanced Pt uptake in *Chlorella fusca* and *Chlamydomonas reinhardtii* . These discrepancies suggest that factors such as DOC composition and water chemistry may critically influence metal bioavailability.

Consistent with its effect on bioaccumulation, DOC also mitigated Pd‐induced mortality in *D. magna* . No mortality occurred between 1 and 8 mg C/L DOC, whereas at 0.8 mg C/L, Pd toxicity was reduced (LC_50_ = 74 ± 1 μg/L) compared to conditions without DOC (LC_50_ = 52 ± 2 μg/L) (Figure 2). This protective trend mirrors findings for other metals such as Cu. Al‐Reasi et al. (2012) reported a DOC‐driven increase in Cu LC_50_ from 19 ± 5 μg/L to values between 26 and 160 μg/L, whereas Macoustra et al. (2020) observed EC_50_ values for Cu in *Chlorella* rising from 1.9 to 63 μg/L as DOC concentrations increased. In contrast, Hourtané et al. (2022) noted increased Pt toxicity in the presence of humic acids, highlighting again the importance of DOC quality and metal‐specific interactions.

Thermodynamic modeling using Visual MINTEQ revealed that Pd (OH)_2_(aq) was the dominant species under exposure conditions (99%). WHAM simulations further indicated that DOC, particularly fulvic acids, strongly complexed Pd, thereby reducing the concentration of free, bioavailable species. These findings are supported by bioaccumulation data (Figure 6), which show a significant inverse correlation between the fraction of Pd bound to fulvic acids and Pd accumulation in *D. magna* . The reduction in bioavailability and toxicity, therefore, is primarily driven by complexation rather than precipitation, as evidenced by the positive correlation between bioaccumulated Pd and modeled Pd (OH)_2_ concentrations. To assess Pd uptake kinetics, an uptake rate constant (*k*_*uw*_) of 0.014 L/g/d was estimated, aligning with values reported for metals exhibiting limited waterborne assimilation (e.g., Se: 0.18 L/g/day). In contrast, the highly unrealistic *k*_*uw*_ value of 2 × 10^8^ L/g/day calculated for free Pd^2+^ reinforces the hypothesis that Pd (OH)_2_ is the primary bioavailable species. Although this estimate may be conservative due to potential Pd losses during the 48‐h exposure, it highlights the importance of chemical speciation in understanding uptake dynamics. Notably, estimates of Pd complexation derived from LC_50_ data corroborate model predictions: At 0.8 mg C/L, 28% of Pd was estimated to be complexed, consistent with WHAM projections, indicating that 88% of Pd is bound to fulvic acids at 1.4 mg C/L (Figure S1). The observed correlation between pH and Pd concentrations (Table S1) further supports the influence of DOC on Pd speciation. Without DOC, higher pH favors the formation of soluble Pd (OH)_2_(aq), enhancing Pd availability. In contrast, in DOC‐rich conditions, elevated pH promotes deprotonation of functional groups on organic ligands, strengthening Pd complexation and reducing the concentration of dissolved Pd (OH)_2_. These pH‐dependent trends are consistent with WHAM simulations and highlight the complex interplay between pH and DOC in regulating metal speciation. As noted by Stockdale et al. (2011), such interactions are key to understanding trace metal bioavailability, though the reliance on empirical constants in speciation models necessitates experimental validation to improve predictive accuracy.

Importantly, the DOC concentrations tested in this study (0.5–8 mg C/L) fall within environmentally relevant ranges, offering a realistic assessment of their potential protective effects. Long‐term monitoring across Eastern Canada has reported DOC concentrations ranging from approximately 1.8 to 16.2 mg/L in freshwater lakes, with mean values between 3.7 and 9.0 mg/L depending on the region (Zhang et al. 2009; Couture et al. 2012; Imtiaz et al. 2020). These values highlight the natural variability of DOC in oligotrophic and mesotrophic systems, confirming the ecological relevance of our experimental conditions. Although minor discrepancies between empirical data and model outputs may reflect differences in DOC composition or model limitations, the overall findings clearly demonstrate that Pd–DOC complexation plays a critical role in mitigating both bioaccumulation and toxicity. These insights are essential for improving environmental risk assessments of Pd and for informing the development of water quality guidelines for this emerging contaminant.

### 4.4 Chronic Toxicity of Pd on Reproduction

The chronic exposure of *D. magna* neonates to Pd until adulthood significantly affected reproductive performance. A significant reduction in reproductive parameters was observed compared to controls (Figure 3), except for the timing of the first brood (Figure S2). These findings indicate heightened sensitivity of *D. magna* reproduction to Pd exposure. This reproductive decline may be attributed to Pd‐induced stress, impairing the health of *D. magna* and reducing viable offspring numbers. The absence of a significant difference in the timing of the first brood indicates that the initial health of *D. magna* was maintained, but their physiological condition deteriorated progressively with prolonged exposure. The reduced number of offspring and broods may also stem from inhibited growth, as Pd‐exposed daphnids were significantly smaller than controls, confirmed by lower dry weights. This growth inhibition aligns with previous findings in *D. magna* exposed to Cd (1 and 5 μg/L) over 14 days (Bodar et al. 1988).

It should be noted that the duration of chronic exposure was reduced from 21 to 15 days due to high parental mortality at elevated Pd concentrations. Although this adjustment may limit direct comparison with studies adhering to the standard 21‐day protocol, *D. magna* typically produces multiple broods within the first 2 weeks of life, releasing a new brood every 3–4 days. Therefore, a 15‐day exposure remains ecologically relevant for assessing sublethal endpoints. Moreover, the consistent, dose‐dependent reduction in reproductive output observed during this period suggests that the toxic effects of Pd were already well established. Nonetheless, potential long‐term effects emerging beyond 15 days could not be evaluated and remain to be addressed in future studies using the full 21‐day protocol. These reproductive and growth impairments may have broader ecological implications. Reduced fecundity and smaller body size in *Daphnia* could decrease population resilience and increase susceptibility to predation, potentially disrupting freshwater food webs and ecosystem functioning (Lynch 1980; Rodrigues et al. 2020).

Despite limited data on chronic PGE toxicity in aquatic organisms, Borgmann et al. (2005) reported a nominal 7‐day LC_50_ of 570 mg/L for Pd in *Hyalella azteca* . Schertzinger et al. (2017) demonstrated that reproduction was the most sensitive endpoint in *C. elegans* , with effects observed from 10 mg/L and near‐complete inhibition between 200 and 800 mg/L. Fertility decreased from 100 to 200 mg/L, associated with growth inhibition. Pd emerged as the most toxic PGE (Pd > Pt > Rh), underscoring its reproductive toxicity. Comparable effects have been observed with other class B metals. Chronic exposure to Cd and Cu(I)—which tend to accumulate intracellularly—also impairs *D. magna* reproduction. Pérez and Hoang (2017) found that Cd at 1.5 μg/L significantly reduced viable neonates and reproductive rates. Zn, as a borderline metal, only affected neonate viability at high concentrations but modulated Cd's effects depending on its own concentration. Martins et al. (2017) observed reduced growth and reproduction (i.e., decreasing offspring production and delayed reproduction) in *Daphnia longispina* after exposure to Cu (≥ 150 μg/L) and Zn (≥ 500 μg/L).

These effects are often due to metals interfering with essential physiological processes like protein synthesis and hormonal regulation, leading to oxidative stress, cellular damage, and impaired reproduction (Choudhary et al. 2007; Kim et al. 2017; Mondal et al. 2018; Paschoalini and Bazzoli 2021). For instance, Pd exposure over a 4‐week period has been associated with DNA damage in Sprague–Dawley rats (via gavage) and *Sphagnum magellanicum* plants (in nutrient medium), with effects observed across a dose range of 0.1–10 mg/g, the highest dose causing the most severe genotoxicity (Gagnon et al. 2006). Beyond genotoxicity, chronic Pd exposure disrupts multiple physiological pathways. In a 10‐week experiment, *D. polymorpha* mussels exposed to nominal Pd^2+^ concentrations of 0.05, 5, 50, and 500 μg/L under fasting conditions exhibited significant Pd bioaccumulation, along with a marked induction of metallothioneins. This response suggests a disruption of metal homeostasis and activation of cellular detoxification mechanisms (Frank et al. 2008). In parallel, mussels exposed to the highest Pd^2+^ concentration (500 μg/L) showed a substantial increase in the expression of heat shock protein 70 (hsp70), reaching levels 25 times higher than in the control group. This response far exceeded those induced by Cd^2+^ (sixfold) and Pb^2+^ (12‐fold), indicating pronounced cellular stress and protein misfolding (Singer et al. 2005). These findings highlight the multifaceted nature of Pd toxicity, encompassing DNA damage, oxidative stress, and proteotoxicity. Further investigation into how these molecular disruptions affect *D. magna* reproduction is crucial for refining the ecotoxicological risk assessment of Pd in freshwater environments.

Weight‐normalized oxygen consumption rate in *D. magna* exposed to Pd for 15 days did not differ significantly from controls (Figure 4), emphasizing the importance of considering organismal biomass in respirometric analyses. As oxygen consumption scales with biomass (Killen et al. 2021), failing to normalize for weight may confound results. The absence of a respiratory effect under these conditions suggests that Pd does not impair metabolic function at tested concentrations. Because reproduction emerged as the most sensitive endpoint, further mechanistic studies are warranted to clarify Pd's broader physiological impacts. Such investigations would enhance ecological risk assessments and improve predictive toxicological modeling of Pd exposure.

## 5 Conclusion

This study examines the acute toxicity of Pd on *D. magna* by determining the EC_50_ and LC_50_ and highlights the essential role of natural organic matter. In the absence of DOC, Pd demonstrates moderate acute toxicity compared to other metals. However, the presence of DOC significantly diminishes Pd bioaccumulation and toxicity by forming complexes that reduce its bioavailability. These results underscore the importance of DOC in modulating Pd toxicity and emphasize the need to integrate DOC considerations into ecotoxicological evaluations. Chronic exposure impacts reproduction and growth, with bioaccumulation proportional to Pd concentrations measured in the exposure medium. Such assessments are particularly crucial for reflecting prolonged contaminant exposure in natural ecosystems. The observed declines in reproduction point to potential disruptions in population dynamics and ecological balance.

The limited knowledge about the toxicity of PGE, particularly Pd, necessitates the generation of more data for different organisms to build SSD relationships used to derive guidelines. Despite low environmental concentrations currently reported in the literature, increasing demand and usage may raise these levels in the future. Establishing environmental standards and water quality guidelines is essential. Future studies should consider measured exposure concentrations, chemical speciation, and DOC effects, adhering to established protocols to ensure data reliability for ecological risk assessment.

## Author Contributions

**Rania Boukhari:** writing – original draft, writing – review and editing, conceptualization, data curation, formal analysis, visualization, methodology, investigation, software. **Dominic E. Ponton:** conceptualization, writing – review and editing, methodology, investigation. **Maikel Rosabal:** conceptualization, writing – review and editing, funding acquisition, supervision. **Kristin K. Mueller:** conceptualization, funding acquisition, writing – review and editing. **Marc Amyot:** conceptualization, writing – review and editing, methodology, supervision, funding acquisition, project administration.

## Conflicts of Interest

The authors declare no conflicts of interest.

## Supporting information


**Table S1** pH Values (mean ± standard deviation) in acute and chronic toxicity tests.
**Figure S1** Estimations of the fraction of Pd bound to Fulvic Acid (FA) (%) obtained using the thermodynamic software WHAM7.
**Figure S2** Day of first egg laying in *D. magna* as a function of measured Pd concentrations in water (measured [Pd] in water μg/L). Values represent the mean ± standard error (*n* = 10).
**Figure S3** Non‐normalized oxygen consumption rate in adult *D. magna* after 15 days of exposure as a function of nominal Pd concentrations in water (μg O_2_/h) (*n* = 7 for 0 μg/L; *n* = 6 for 2 μg/L; *n* = 7 for 5 μg/L and 10 μg/L; *n* = 2 for 20 μg/L). Linear model is significant (*p* < 0.05).
**Figure S4** Concentration‐response curves demonstrating the effect of Pd on the mortality of *D. magna* over a 48‐hour exposure period in the presence (a) and absence (b) of dissolved organic carbon (DOC) (*n* = 4). Each dot represents the percentage of mortality within a replicate. In the presence of DOC (b), the measured DOC concentration was 0.8 mg C/L. The medium used was *Daphnia* culture water without M4 medium. Shaded areas represent the 95% confidence interval.
**Figure S5** (a) Total number of offspring per *D. magna* (*n* = 10 for 0 μg/L; *n* = 9 for 2 and 5 μg/L; *n* = 8 for 10 μg/L; *n* = 7 for 20 μg/L; *n* = 8 for 24 μg/L; *n* = 5 for 26 μg/L), (b) Total number of broods per *D. magna* (*n* = 10), (c) Dry weight of the parent of *D. magna* (mg) (*n* = 10 for 0 μg/L; *n* = 7 for 2 μg/; *n* = 8 for 5 μg/L; *n* = 2 for 10 μg/L; *n* = 1 for 20 μg/L), significant differences (*p* < 0.05) are indicated by distinct letters, (d) Survival of the parent of *D. magna* (*n* = 10) as a function of measured Pd concentrations in water (Nominal [Pd] water, μg/L). The values represent mean ± standard error. Non‐Linear regressions were fitted using the drm function in the drc package (R): a four‐parameter log‐logistic model (LL.4) for panel (a), and three‐parameter log‐logistic models (LL.3) for panels (b) and (d) (all models significant at *p* < 0.05).
**Figure S6:** Oxygen consumption rate in adult *D. magna* after 15 days of exposure as a function of nominal Pd concentrations in water, standardized per mg dw of daphnid biomass (μg O_2_/mg dw/h) (*n* = 7 for 0 μg/L; *n* = 6 for 2 μg/L; *n* = 7 for 5 μg/L and 10 μg/L; *n* = 2 for 20 μg/L). Linear model is significant (*p* < 0.05).
**Figure S7** Pd bioaccumulation ([Pd] μg/g dry weight) in *D. magna* as a function of nominal Pd concentrations in water (Nominal [Pd] in water μg/L). Panel (a): acute exposure (48 h), each dot represents a pooled sample of 10 individuals per concentration. Panel (b): chronic exposure (15 days), each dot represents an individual surviving adult. Linear models are significant (*p* < 0.05).

## Data Availability

The data that support the findings of this study are available from the corresponding author upon reasonable request.

## References

[jat4854-bib-0001] Adams, W. , R. Blust , R. Dwyer , et al. 2020. “Bioavailability Assessment of Metals in Freshwater Environments: A Historical Review.” Environmental Toxicology and Chemistry 39, no. 1: 48–59. 10.1002/etc.4558. 31880839 PMC11382335

[jat4854-bib-0002] Agilent . 2022. “Agilent *ICP‐QQQ* Applications in Geochemistry, Mineral Analysis and Nuclear Science.”

[jat4854-bib-0003] Al‐Reasi, H. A. , D. Scott Smith , and C. M. Wood . 2012. “Evaluating the Ameliorative Effect of Natural Dissolved Organic Matter (DOM) Quality on Copper Toxicity to *Daphnia magna*: Improving the BLM.” Ecotoxicology 21: 524–537. 10.1007/s10646-011-0813-z. 22072428

[jat4854-bib-0004] Barbieri, E. 2009. “Effects of Zinc and Cadmium on Oxygen Consumption and Ammonium Excretion in Pink Shrimp (*Farfantepenaeus paulensis*, Pérez‐Farfante, 1967, Crustacea).” Ecotoxicology (London, England) 18, no. 3: 312–318. 10.1007/s10646-008-0285-y. 19031115

[jat4854-bib-0005] Batley, G. E. , and P. G. C. Campbell . 2022. “Metal Contaminants of Emerging Concern in Aquatic Systems.” Environmental Chemistry 19, no. 1: 23–40. 10.1071/EN22030.

[jat4854-bib-0006] Bluteau, G. , D. E. Ponton , M. Rosabal , and M. Amyot . 2025. “Biodynamics and Environmental Concentrations of the Platinum Group Elements in Freshwater Systems.” Environmental Science & Technology 59, no. 12: 6203–6213. 10.1021/acs.est.4c08750. 40118076 PMC11966755

[jat4854-bib-0007] Bodar, C. W. M. , C. J. van Leeuwen , P. A. Voogt , and D. I. Zandee . 1988. “Effect of Cadmium on the Reproduction Strategy of *Daphnia magna* .” Aquatic Toxicology 12, no. 4: 301–309. 10.1016/0166-445X(88)90058-6.

[jat4854-bib-0008] Borgmann, U. , Y. Couillard , P. Doyle , and D. G. Dixon . 2005. “Toxicity of Sixty‐Three Metals and Metalloids to *Hyalella azteca* at Two Levels of Water Hardness.” Environmental Toxicology and Chemistry 24, no. 3: 641–652. 10.1897/04-177R.1. 15779765

[jat4854-bib-0009] Bryan, S. E. , E. Tipping , and J. Hamilton‐Taylor . 2002. “Comparison of Measured and Modelled Copper Binding by Natural Organic Matter in Freshwaters.” Comparative Biochemistry and Physiology, Part C: Toxicology & Pharmacology 133, no. 1–2: 37–49. 10.1016/S1532-0456(02)00083-2. 12356515

[jat4854-bib-0010] Centre d'Expertise en Analyse Environnementale du Québec . 2021. “Détermination de la Toxicité: Létalité (CL50 48 h) Chez la Daphnie Daphnia magna. MA. 500 – D.mag. 1.1, Rév. 3, Ministère de l'Environnement et de la Lutte Contre les Changements Climatiques du Québec.” https://www.ceaeq.gouv.qc.ca/methodes/pdf/methode‐analyse‐500‐daphnia‐magna.pdf.

[jat4854-bib-0011] Chen, M. , S. Chen , M. Du , et al. 2015. “Toxic Effect of Palladium on Embryonic Development of Zebrafish.” Aquatic Toxicology 159: 208–216. 10.1016/j.aquatox.2014.12.015. 25550166

[jat4854-bib-0012] Cho, H. , C. S. Ryu , S. A. Lee , et al. 2022. “Endocrine‐Disrupting Potential and Toxicological Effect of Para‐Phenylphenol on *Daphnia magna* .” Ecotoxicology and Environmental Safety 243: 113965. 10.1016/j.ecoenv.2022.113965. 35994907

[jat4854-bib-0013] Choudhary, M. , U. K. Jetley , M. A. Khan , S. Zutshi , and T. Fatma . 2007. “Effect of Heavy Metal Stress on Proline, Malondialdehyde, and Superoxide Dismutase Activity in the Cyanobacterium *Spirulina platensis*‐S5.” Ecotoxicology and Environmental Safety 66, no. 2: 204–209. 10.1016/j.ecoenv.2006.02.002. 16600377

[jat4854-bib-0014] Cicchella, D. , B. de Vivo , and A. Lima . 2003. “Palladium and Platinum Concentration in Soils From the Napoli Metropolitan Area, Italy: Possible Effects of Catalytic Exhausts.” Science of the Total Environment 308, no. 1–3: 121–131. 10.1016/S0048-9697(02)00632-0. 12738206

[jat4854-bib-0015] Cocherell, S. A. , D. E. Cocherell , G. J. Jones , et al. 2011. “ *Oncorhynchus mykiss* Energetic Responses to Pulsed Flows in the American River, California, Assessed by Electromyogram Telemetry.” Environmental Biology of Fishes 90: 29–41. 10.1007/s10641-010-9714-x.

[jat4854-bib-0016] Couture, S. , D. Houle , and C. Gagnon . 2012. “Increases of Dissolved Organic Carbon in Temperate and Boreal Lakes in Quebec, Canada.” Environmental Science and Pollution Research 19, no. 2: 361–371. 10.1007/s11356-011-0565-6. 21755324

[jat4854-bib-0017] Dang, D. H. , W. Wang , D. Omanović , and A. Mucci . 2024. “Mixing Behaviour and Sources of Ag, Pd, and Other Trace Elements in the Estuary and Gulf of St. Lawrence Under Winter Conditions.” Chemosphere 363: 142935. 10.1016/j.chemosphere.2024.142935. 39053777

[jat4854-bib-0018] de Medeiros, A. M. Z. , F. Côa , O. L. Alves , D. S. T. Martinez , and E. Barbieri . 2020. “Metabolic Responses in *Geophagus iporangensis* to Graphene Oxide and Trace Elements.” Chemosphere 243: 125316. 10.1016/j.chemosphere.2019.125316. 31733537

[jat4854-bib-0019] Duffus, J. H. 2002. ““Heavy Metals”—A Meaningless Term? (IUPAC Technical Report.” Pure and Applied Chemistry 74, no. 5: 793–807. 10.1351/pac200274050793.

[jat4854-bib-0020] Duro, L. , M. Grivé , E. Cera , C. Domènech , and J. Bruno . 2006. “Update of a Thermodynamic Database for Radionuclides to Assist Solubility Limits Calculation for Performance Assessment (December 2006). Enviros Spain, S.L., Technical Report for SKB.”

[jat4854-bib-0021] Ebert, D. 2005. Ecology, Epidemiology, and Evolution of Parasitism in Daphnia [Internet]. National Library of Medicine (US), National Center for Biotechnology Information. http://www.ncbi.nlm.nih.gov/entrez/query.fcgi?db=Books.

[jat4854-bib-0022] Elendt, B. P. , and W. R. Bias . 1990. “Trace Nutrient Deficiency in *Daphnia magna* Cultured in Standard Medium for Toxicity Testing. Effects of the Optimization of Culture Conditions on Life History Parameters of *D. magna* .” Water Research 24, no. 9: 1157–1167. 10.1016/0043-1354(90)90180-E.

[jat4854-bib-0023] Fortin, C. F. Wang , and D. Pitre . 2011. “Critical Review of Platinum Group Elements (Pd, Pt, Rh) in Aquatic Ecosystems.” Research Report No. R‐1269, 47.

[jat4854-bib-0024] Frank, S. N. , C. Singer , and B. Sures . 2008. “Metallothionein (MT) Response Following Long‐Term Palladium Exposure in Zebra Mussels, *Dreissena polymorpha* .” Environmental Research 108, no. 3: 309–314. 10.1016/j.envres.2008.07.021. 18762294

[jat4854-bib-0025] Fujiwara, K. , Y. Matsumoto , H. Kawakami , M. Aoki , and M. Tuzuki . 2008. “Evaluation of Metal Toxicity in *Chlorella kessleri* From the Perspective of the Periodic Table.” Bulletin of the Chemical Society of Japan 81, no. 4: 478–488. 10.1246/bcsj.81.478.

[jat4854-bib-0026] Gagnon, Z. E. , C. Newkirk , and S. Hicks . 2006. “Impact of Platinum Group Metals on the Environment: A Toxicological, Genotoxic and Analytical Chemistry Study.” Journal of Environmental Science and Health, Part A 41, no. 3: 397–414. 10.1080/10934520500423592. 16484072

[jat4854-bib-0027] Government of Canada . 2024. “Critical Minerals: A Canadian Opportunity.” https://www.canada.ca/en/campaign/critical‐minerals‐in‐canada/critical‐minerals‐an‐opportunity‐for‐canada.html.

[jat4854-bib-0028] Hourtané, O. , G. Rioux , P. G. C. Campbell , and C. Fortin . 2022. “Algal Bioaccumulation and Toxicity of Platinum Are Increased in the Presence of Humic Acids.” Environmental Chemistry 19, no. 4: 144–155. 10.1071/EN22037.

[jat4854-bib-0029] Hu, Q. , X. Yang , Z. Huang , J. Chen , and G. Yang . 2005. “Simultaneous Determination of Palladium, Platinum, Rhodium, and Gold via On‐Line Solid‐Phase Extraction Coupled With High‐Performance Liquid Chromatography Using 5‐(2‐Hydroxy‐5‐Nitrophenylazo) Thiorhodanine as a Pre‐Column Derivatization Reagent.” Journal of Chromatography A 1094, no. 1–2: 77–82. 10.1016/j.chroma.2005.07.090. 16257292

[jat4854-bib-0030] Hylton, C. A. , and M. T. K. Tsui . 2021. “Modification of Acute Toxicity of Inorganic Mercury and Methylmercury to *Daphnia magna* by Dietary Addition.” Scientific Reports 11: 22865. 10.1038/s41598-021-02300-4. 34819591 PMC8613259

[jat4854-bib-0031] Imtiaz, M. N. , A. M. Paterson , S. N. Higgins , H. Yao , S. Couture , and J. J. Hudson . 2020. “Dissolved Organic Carbon in Lakes of Eastern Canada: New Models and Relationships With Regional and Global Factors.” Science of the Total Environment 726: 138400. 10.1016/j.scitotenv.2020.138400. 32315845

[jat4854-bib-0032] Khangarot, B. S. 1991. “Metal Toxicity to the Freshwater Oligochaete *Tubifex tubifex* (Müller).” Bulletin of Environmental Contamination and Toxicology 46, no. 6: 901–908. 10.1007/BF01689737. 1863799

[jat4854-bib-0033] Khangarot, B. S. , and S. Das . 2009. “Acute Toxicity of Metals and Reference Toxicants to the Freshwater Ostracod *Cypris subglobosa* Sowerby, 1840, and Correlation With EC50 Values of Other Test Organisms.” Journal of Hazardous Materials 172, no. 2–3: 641–649. 10.1016/j.jhazmat.2009.07.038. 19683870

[jat4854-bib-0034] Killen, S. S. , E. A. F. Christensen , D. Cortese , et al. 2021. “Guidelines for Reporting Methods to Estimate Metabolic Rates by Aquatic Intermittent‐Flow Respirometry.” Journal of Experimental Biology 224, no. 18: jeb242522. 10.1242/jeb.242522. 34520540 PMC8467026

[jat4854-bib-0035] Kim, H. , B. Yim , C. Bae , and Y. M. Lee . 2017. “Acute Toxicity and Antioxidant Responses in *Daphnia magna* Exposed to Cadmium, Lead, Mercury, Bisphenol A, and 4‐Nonylphenol.” Toxicology and Environmental Health Sciences 9, no. 1: 41–49. 10.1007/s13530-017-0302-8.

[jat4854-bib-0036] Kolts, J. M. , M. L. Brooks , B. D. Cantrell , C. J. Boese , R. A. Bell , and J. S. Meyer . 2008. “Dissolved Fraction of Standard Laboratory Cladoceran Food Alters Toxicity of Waterborne Silver to *Ceriodaphnia dubia* .” Environmental Toxicology and Chemistry 27, no. 6: 1426–1434. 10.1897/07-326.1. 18220444

[jat4854-bib-0037] Ladonin, D. V. 2018. “Distribution of Platinum‐Group Elements in Soils and Street Dust From the Southeastern Administrative District of Moscow.” Eurasian Soil Science 51, no. 3: 268–276. 10.1134/S1064229318030055.

[jat4854-bib-0038] le Faucheur, S. , J. Mertens , E. van Genderen , A. Boullemant , C. Fortin , and P. G. C. Campbell . 2021. “Development of Quantitative Ion Character–Activity Relationship (ICAR) Models to Address Data Gaps for Technology‐Critical Elements.” Environmental Toxicology and Chemistry 40, no. 4: 1139–1148. 10.1002/etc.4960. 33315280

[jat4854-bib-0040] Liu, Y. , F. Ding , L. Zhang , et al. 2021. “Urban Palladium Transport and Modeling: A Case Study in Haikou, China.” Urban Water Journal 18, no. 1: 25–32. 10.1080/1573062X.2020.1850805.

[jat4854-bib-0041] Lynch, M. 1980. “The Evolution of Cladoceran Life Histories.” Quarterly Review of Biology 55, no. 1: 23–42. 10.1086/411614.

[jat4854-bib-0042] Macoustra, G. K. , D. F. Jolley , J. Stauber , D. J. Koppel , and A. Holland . 2020. “Copper Toxicity Reduction in a Tropical Freshwater Microalga: Influence of Dissolved Organic Matter Source and Seasonality.” Environmental Pollution 266: 115141. 10.1016/j.envpol.2020.115141. 32659625

[jat4854-bib-0043] Martins, C. , F. T. Jesus , and A. J. A. Nogueira . 2017. “Effects of Copper and Zinc on Survival, Growth, and Reproduction of *Daphnia longispina*: Revisiting a Classic Ecotoxicological Model.” Ecotoxicology 26, no. 9: 1157–1169. 10.1007/s10646-017-1841-0. 28828683

[jat4854-bib-0044] Matodzi, V. , M. A. Legodi , and N. T. Tavengwa . 2020. “Platinum Group Metals in Dust, Water, Soil, and Sediments Near a Cement Production Site.” SN Applied Sciences 2, no. 6: 1090. 10.1007/s42452-020-2882-1.

[jat4854-bib-0045] Moldovan, M. , S. Rauch , M. Gómez , M. A. Palacios , and G. M. Morrison . 2001. “Bioaccumulation of Pd, Pt, and Rh by the Freshwater Isopod *Asellus aquaticus* From Urban Particulates and Sediments.” Water Research 35, no. 17: 4175–4183. 10.1016/S0043-1354(01)00136-1. 11791847

[jat4854-bib-0046] Mondal, K. , S. Ghosh , and S. Haque . 2018. “Contamination, Bioaccumulation, and Toxicological Impacts of Cadmium, Mercury, and Lead on Freshwater Fish: A Review.” International Journal of Zoology Studies 3, no. 2: 153–159.

[jat4854-bib-0047] Nikinmaa, M. , E. Suominen , and K. Anttila . 2019. “Water‐Soluble Crude Oil Fractions Induce Variability and Transgenerational Effects in *Daphnia magna* .” Aquatic Toxicology 211: 137–140. 10.1016/j.aquatox.2019.04.004. 30978588

[jat4854-bib-0048] Odiyo, J. O. , H. M. Bapela , R. Mugwedi , and L. Chimuka . 2005. “Trace and Platinum Group Metals in Environmental Media in Thohoyandou, South Africa.” Water SA 31, no. 4: 581–588. 10.4314/wsa.v31i4.5148.

[jat4854-bib-0049] OECD . 2004. Test No. 202: *Daphnia* sp. Acute Immobilisation Test (OECD Guidelines for the Testing of Chemicals). OECD Publishing. https://www.oecd.org/en/publications/test‐no‐202‐daphnia‐sp‐acute‐immobilisation‐test_9789264069947‐en.html.

[jat4854-bib-0050] OECD . 2012. Test No. 211: *Daphnia Magna* Reproduction Test (OECD Guidelines for the Testing of Chemicals). OECD Publishing. https://www.oecd.org/en/publications/test‐no‐211‐daphnia‐magna‐reproduction‐test_9789264185203‐en.html.

[jat4854-bib-0051] Okamoto, A. , M. Yamamuro , and N. Tatarazako . 2015. “Acute Toxicity Screening of 50 Metals Using *Daphnia magna* .” Journal of Applied Toxicology 35, no. 7: 824–830. 10.1002/jat.3078. 25382633

[jat4854-bib-0052] Paschoalini, A. L. , and N. Bazzoli . 2021. “Impacts of Heavy Metals on Neotropical Freshwater Fish: A Decade of Ecotoxicological Research.” Aquatic Toxicology 237: 105906. 10.1016/j.aquatox.2021.105906. 34246836

[jat4854-bib-0053] Pérez, E. , and T. C. Hoang . 2017. “Chronic Toxicity of Binary‐Metal Mixtures of Cadmium and Zinc to *Daphnia magna* .” Environmental Toxicology and Chemistry 36, no. 10: 2739–2749. 10.1002/etc.3830. 28430390

[jat4854-bib-0054] R Core Team . 2024. R: A Language and Environment for Statistical Computing. R Foundation for Statistical Computing. https://www.R‐project.org/.

[jat4854-bib-0055] Rauch, S. , H. F. Hemond , and B. Peucker‐Ehrenbrink . 2004. “Temporal Changes in PGE Concentrations and Os Isotope Ratios in Urban Lake Sediments.” Environmental Science & Technology 38, no. 2: 396–402. 10.1021/es0347686. 14750713

[jat4854-bib-0056] Ritz, C. , F. Baty , J. C. Streibig , and D. Gerhard . 2015. “Dose‐Response Analysis Using R.” PLoS ONE 10, no. 12: e0146021. 10.1371/journal.pone.0146021. 26717316 PMC4696819

[jat4854-bib-0057] Rodrigues, G. Z. P. , M. Finkler , A. L. H. Garcia , and C. E. da Rosa . 2020. “Evaluation of Transgenerational Effects Caused by Metals as Environmental Pollutants in *Daphnia magna* .” Environmental Monitoring and Assessment 192: 755. 10.1007/s10661-020-08713-4. 33170361

[jat4854-bib-0058] Rudnick, R. L. , and S. Gao . 2014. “Composition of the Continental Crust.” In Treatise on Geochemistry, edited by H. D. Holland and K. K. Turekian , vol. 4, 2nd ed., 1–51. Elsevier. 10.1016/B978-0-08-095975-7.00301-6.

[jat4854-bib-0059] Savignan, L. , S. Faucher , P. Chéry , and G. Lespes . 2021. “Current State of Platinum Group Element Contamination in Soils: A Critical Review.” Chemosphere 271: 129517. 10.1016/j.chemosphere.2020.129517. 33450423

[jat4854-bib-0060] Sawasdee, B. , and H.‐R. Köhler . 2010. “Metal Sensitivity During Embryogenesis of the Freshwater Snail *Marisa cornuarietis* .” Ecotoxicology 19, no. 8: 1487–1495. 10.1007/s10646-010-0534-8. 20711673

[jat4854-bib-0061] Schertzinger, G. , S. Zimmermann , D. Grabner , and B. Sures . 2017. “Chronic Exposure to Pd, Pt, and Rh: Sublethal Effects on *Caenorhabditis elegans* .” Environmental Pollution 230: 31–39. 10.1016/j.envpol.2017.06.040. 28644982

[jat4854-bib-0062] Singer, C. , S. Zimmermann , and B. Sures . 2005. “Induction of Heat Shock Proteins (hsp70) in the Zebra Mussel (*Dreissena polymorpha*) Following Exposure to Platinum Group Metals (Platinum, Palladium, Rhodium): Comparison With Lead and Cadmium Exposures.” Aquatic Toxicology 75, no. 1: 65–75. 10.1016/j.aquatox.2005.07.004. 16111776

[jat4854-bib-0063] Soyol‐Erdene, T. O. , and Y. Huh . 2012. “Dissolved Platinum in East Asian Rivers: Implications for the Oceanic Budget.” Geochemistry, Geophysics, Geosystems 13, no. 6. 10.1029/2012GC004102.

[jat4854-bib-0064] Spada, N. , A. Bozlaker , and S. Chellam . 2012. “Multi‐Elemental Analysis of Road and Tunnel Dusts by DRC‐ICP‐MS: Emissions of PGEs and Other Metals From Vehicles.” Analytica Chimica Acta 735: 1–8. 10.1016/j.aca.2012.05.026. 22713911

[jat4854-bib-0065] Stockdale, A. , N. D. Bryan , and S. Lofts . 2011. “Estimation of Model VII Humic Binding Constants for Pd^2+^, Sn^2+^, U^4+^, NpO_2_ ^2+^, Pu^4+^, and PuO_2_ ^2+^ .” Journal of Environmental Monitoring 13, no. 10: 2946–2950. 10.1039/C1EM10407A. 21870014

[jat4854-bib-0066] Sures, B. , and S. Zimmermann . 2007. “Impact of Humic Substances on the Aqueous Solubility, Uptake and Bioaccumulation of Platinum, Palladium and Rhodium in Exposure Studies With *Dreissena polymorpha* .” Environmental Pollution 146, no. 2: 444–451. 10.1016/j.envpol.2006.07.004. 17018243

[jat4854-bib-0067] Tang, H. , Z. Peng , R. Tian , et al. 2023. “Platinum‐Group Metals: Demand, Supply, Applications and Their Recycling From Spent Automotive Catalysts.” Journal of Environmental Chemical Engineering 11, no. 5: 110237. 10.1016/j.jece.2023.110237.

[jat4854-bib-0068] Tsui, M. T. K. , and W. X. Wang . 2007. “Biokinetics and Tolerance Development of Toxic Metals in *Daphnia magna* .” Environmental Toxicology and Chemistry 26, no. 5: 1023–1032. 10.1897/06-430R.1. 17521151

[jat4854-bib-0069] US Geological Survey . 2024. “Mineral Commodity Summaries 2024 (P. 137). U.S. Department of the Interior.” 10.3133/mcs2024.

[jat4854-bib-0070] Wei, X. , C. Liu , H. Cao , et al. 2019. “Understanding the Features of PGMs in Spent Ternary Automobile Catalysts for Development of Cleaner Recovery Technology.” Journal of Cleaner Production 239: 118031. 10.1016/j.jclepro.2019.118031.

[jat4854-bib-0071] Whiteley, J. D. , and F. Murray . 2005. “Autocatalyst‐Derived Platinum, Palladium and Rhodium (PGE) in Infiltration Basin and Wetland Sediments Receiving Urban Runoff.” Science of the Total Environment 341, no. 1–3: 199–209. 10.1016/j.scitotenv.2004.09.030. 15833252

[jat4854-bib-0072] Wiseman, C. L. S. , Z. Hassan Pour , and F. Zereini . 2016. “Platinum Group Element and Cerium Concentrations in Roadside Environments in Toronto, Canada.” Chemosphere 145: 61–67. 10.1016/j.chemosphere.2015.11.056. 26688240

[jat4854-bib-0073] Witeska, M. , E. Kondera , J. Lipionoga , and A. Jastrzebska . 2010. “Changes in Oxygen Consumption Rate and Red Blood Parameters in Common Carp *Cyprinus carpio* L. After Acute Copper and Cadmium Exposures.” Fresenius Environmental Bulletin 19, no. 1: 115–122.

[jat4854-bib-0074] Wood, S. A. , and D. Vlassopoulos . 1990. “The Dispersion of Pt, Pd and Au in Surficial Media About Two PGE‐Cu‐Ni Prospects in Quebec.” Canadian Mineralogist 28, no. 3: 649–663.

[jat4854-bib-0075] Wren, M. , and Z. E. Gagnon . 2014. “A Histopathological Study of Hudson River Crayfish, *Orconectes virilis*, Exposed to Platinum Group Metals.” Journal of Environmental Science and Health, Part A 49, no. 2: 135–145. 10.1080/10934529.2013.838836. 24171412

[jat4854-bib-0076] Yashchenko, V. , E. I. Fossen , Ø. N. Kielland , and S. Einum . 2016. “Negative Relationships Between Population Density and Metabolic Rates Are Not General.” Journal of Animal Ecology 85, no. 4: 1070–1077. 10.1111/1365-2656.12515. 26970102

[jat4854-bib-0077] Zhang, C. , F. Li , and J. Xiang . 2014. “Acute Effects of Cadmium and Copper on Survival, Oxygen Consumption, Ammonia‐N Excretion, and Metal Accumulation in Juvenile *Exopalaemon Carinicauda* .” Ecotoxicology and Environmental Safety 104: 209–214. 10.1016/j.ecoenv.2014.01.008. 24726930

[jat4854-bib-0078] Zhang, J. , J. Hudson , R. Neal , et al. 2009. “Long‐Term Patterns of Dissolved Organic Carbon in Lakes Across Eastern Canada: Evidence of a Pronounced Climate Effect.” Limnology and Oceanography 55, no. 1: 30–42. 10.4319/lo.2010.55.1.0030.

[jat4854-bib-0079] Zimmermann, S. , F. Alt , J. Messerschmidt , A. von Bohlen , H. Taraschewski , and B. Sures . 2002. “Biological Availability of Traffic‐Related Platinum‐Group Elements (Palladium, Platinum, and Rhodium) and Other Metals to the Zebra Mussel (*Dreissena polymorpha*) in Water Containing Road Dust.” Environmental Toxicology and Chemistry 21, no. 12: 2713–2718. 10.1002/etc.5620211226. 12463569

[jat4854-bib-0080] Zimmermann, S. , C. Wolff , and B. Sures . 2017. “Toxicity of Platinum, Palladium and Rhodium to *Daphnia magna* in Single and Binary Metal Exposure Experiments.” Environmental Pollution 224: 368–376. 10.1016/j.envpol.2017.02.016. 28222978

