# Supplementary material for: Assessing the Acute and Chronic Effects of Palladium on Daphnia magna and the Influence of Natural Organic Matter
Source: J Appl Toxicol. 2025 Jul 10;45(11):2363–77. doi: 10.1002/jat.4854 (PMC12488302; doi:10.1002/jat.4854)
Supplement: Supplementary file 1 — Table S1 pH Values (mean ± standard deviation) in acute and chronic toxicity tests. Figure S1 Estimations of the fraction of Pd bound to Fulvic Acid (FA) (%) obtained using the thermodynamic software WHAM7. Figure S2 Day of first egg laying in D. magna as a function of measured Pd concentrations in water (measured [Pd] in water μg/L). Values represent the mean ± standard error (n = 10). Figure S3 Non‐normalized oxygen consumption rate in adult D. magna after 15 days of exposure as a function of nominal Pd concentrations in water (μg O2/h) (n = 7 for 0 μg/L; n = 6 for 2 μg/L; n = 7 for 5 μg/L and 10 μg/L; n = 2 for 20 μg/L). Linear model is significant (p < 0.05). Figure S4 Concentration‐response curves demonstrating the effect of Pd on the mortality of D. magna over a 48‐hour exposure period in the presence (a) and absence (b) of dissolved organic carbon (DOC) (n = 4). Each dot represents the percentage of mortality within a replicate. In the presence of DOC (b), the measured DOC concentration was 0.8 mg C/L. The medium used was Daphnia culture water without M4 medium. Shaded areas represent the 95% confidence interval. Figure S5 (a) Total number of offspring per D. magna (n = 10 for 0 μg/L; n = 9 for 2 and 5 μg/L; n = 8 for 10 μg/L; n = 7 for 20 μg/L; n = 8 for 24 μg/L; n = 5 for 26 μg/L), (b) Total number of broods per D. magna (n = 10), (c) Dry weight of the parent of D. magna (mg) (n = 10 for 0 μg/L; n = 7 for 2 μg/; n = 8 for 5 μg/L; n = 2 for 10 μg/L; n = 1 for 20 μg/L), significant differences (p < 0.05) are indicated by distinct letters, (d) Survival of the parent of D. magna (n = 10) as a function of measured Pd concentrations in water (Nominal [Pd] water, μg/L). The values represent mean ± standard error. Non‐Linear regressions were fitted using the drm function in the drc package (R): a four‐parameter log‐logistic model (LL.4) for panel (a), and three‐parameter log‐logistic models (LL.3) for panels (b) and (d) (all models significant at p < 0.05). Figure [file JAT-45-2363-s001.docx]

Supplementary Information

Assessing the acute and chronic effects of palladium on *Daphnia magna* and the influence of natural organic matter

Rania Boukhari^1, a^, Dominic E. Ponton^1, a^, Maikel Rosabal^2, a^, Kristin K. Mueller^3^, Marc Amyot^1, a, *^

^1^GRIL, Département des sciences biologiques, Université de Montréal, 375 Ave.Thérèse-Lavoie-Roux, Montréal, QC H2V 0B3, Québec, Canada,

^2^GRIL, Département des sciences biologiques, Université du Québec à Montréal (UQAM), 141 Avenue du Président-Kennedy, Montréal, Québec H2X 1Y4, Canada.

^3^Centre Eau, Terre et Environnement (ETE), Institut national de la recherche scientifique (INRS), 490 rue de la Couronne, Québec, QC G1K 9A9, Canada.

^a^EcotoQ, Centre de recherche en écotoxicologie du Québec, 490 rue de la Couronne, Québec, QC, G1K 9A9, Canada.

* Corresponding author: m.amyot@umontreal.ca

1. **Quality Control of Measured Pd Concentrations by ICP-MS/MS**

Palladium (Pd) concentrations in water samples and biological tissues of *Daphnia magna* were measured using an inductively coupled plasma triple quadrupole mass spectrometer (ICP-MS/MS, Agilent 8900). To enhance analytical precision and minimize matrix interferences, ammonia (NH₃) was used as a reaction gas in the second collision cell. Only Pd concentrations exceeding the detection limit of 0.1 ng/L were reported. To improve accuracy, corrections were applied to account for interference from SrOH⁺ (105), Cd (106, 108) on Pd isotopes (105, 106, 108).

To ensure the accuracy of the results, multiple quality control measures were implemented. Analytical blanks (digestion blanks) were included in each sample batch to monitor potential contamination and reduce analytical bias. Certified reference materials (CRMs), such as OPR2 standards, were used to validate Pd concentrations in water samples, with an average recovery ratios of 103 ± 6% (n = 5).

For the analysis of biological tissues, the CRM IAEA-450 (International Atomic Energy Agency), a reference material derived from a unicellular microalga, was employed. Pd was spiked into this CRM using a high-purity solution to assess the efficiency of digestion and quantification procedures. Recovery ratios for biological samples were 86 ± 13% (n = 3).

The calibration curve for Pd quantification was constructed using certified platinum group element (PGE) standards (SCP Science). Secondary source standards were utilized to verify calibration accuracy, ensuring the reliability of the measured concentrations.

To ensure the reliability of the analytical methods, an intercalibration exercise was performed between ICP-MS instruments at UdeM, partner universities (UQAM and INRS), and the Quebec Ministry of the Environment (MELCCFP), enhancing precision and reproducibility in sample analysis.

**Table S1:** pH Values (mean ± standard deviation) in acute and chronic toxicity tests.

| **Acute test** | | | | **Chronic test** | |
| --- | --- | --- | --- | --- | --- |
| [Pd] Nominal (µg/L) | **pH without DOC** | [Pd] Nominal (µg/L) | **pH With DOC** | [Pd] Nominal (µg/L) | **pH** |
| 0 | 7.3±0.7 | 0 | 7.6±0.1 | 0 | 7.7±0.2 |
| 2 | 7.4±0.8 | 60 | 7.2±0.1 | 5 | 7.8±0.6 |
| 40 | 7.6±0.4 | 80 | 7.5±0.8 | 12 | 8.0±0.1 |
| 80 | 7.4±0.6 | 120 | 6.8±0.4 | 30 | 8.3±0.3 |
| 140 | 7.8±0.8 | 140 | 6.8±0.2 | 48 | 8.2±0.8 |
| 180 | 8.1±0.9 | 180 | 6.6±0.9 | 120 | 8.4±0.5 |


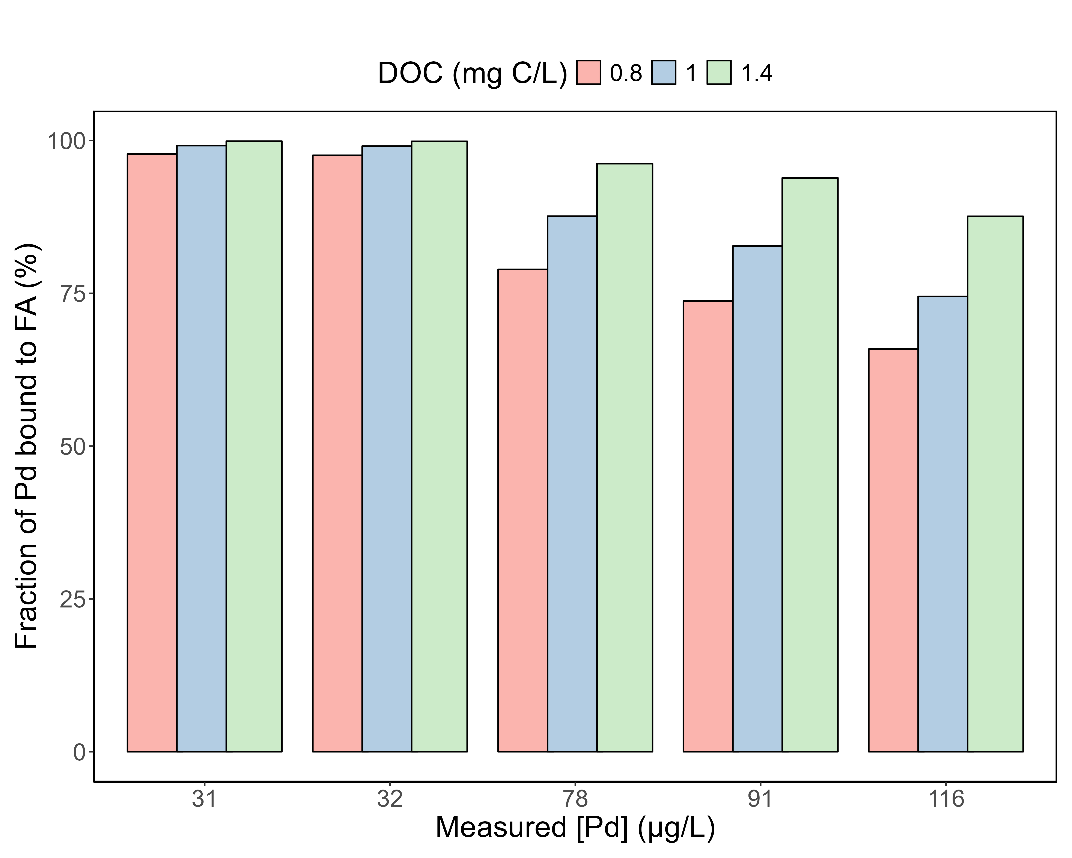


**Figure S1:** Estimations of the fraction of Pd bound to Fulvic Acid (FA) (%) obtained using the thermodynamic software WHAM7.


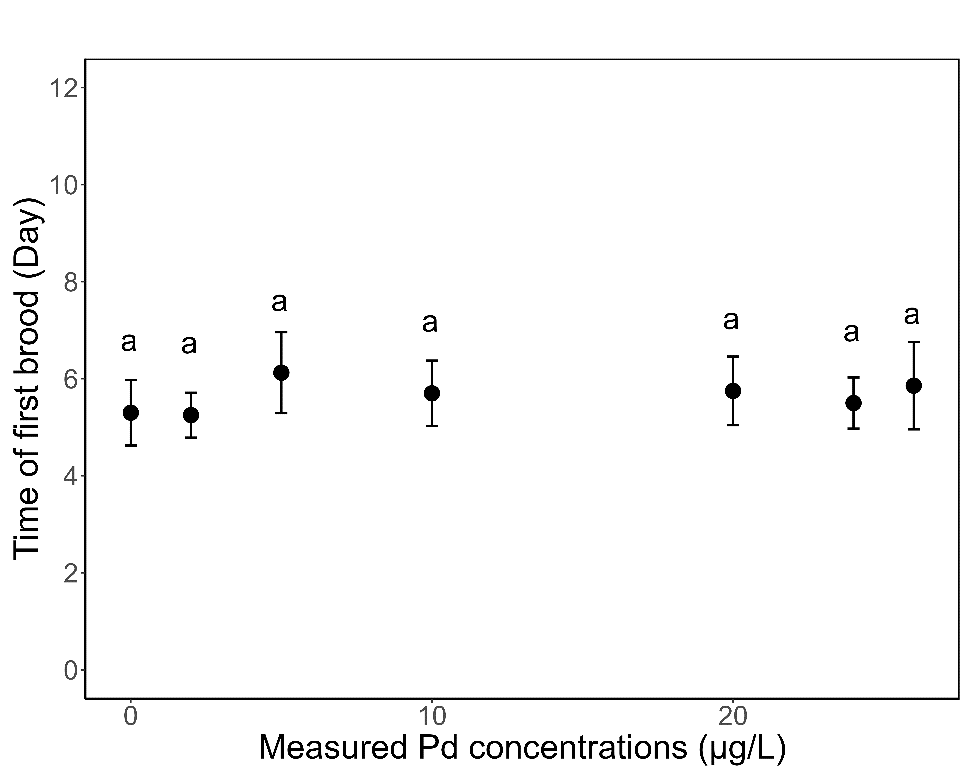


**Figure S2:** Day of first egg laying in *D. magna* as a function of measured Pd concentrations in water (measured [Pd] in water µg/L). Values represent the mean ± standard error (n=10).


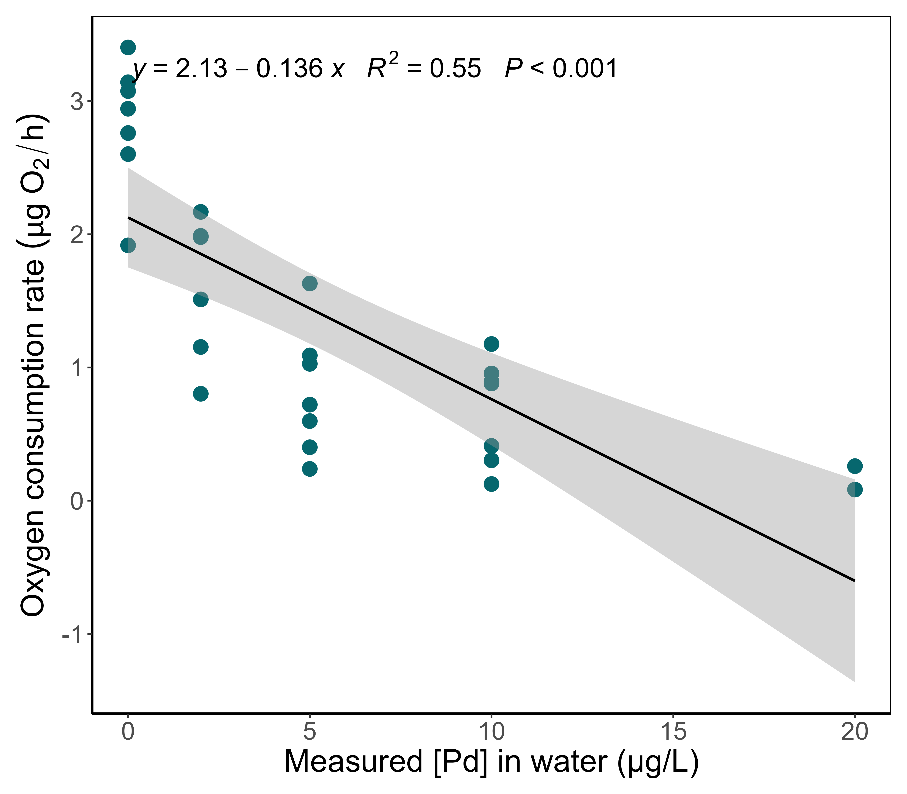


**Figure S3:** Non-normalized oxygen consumption rate in adult *D. magna* after 15 days of exposure as a function of nominal Pd concentrations in water (µg O₂/h) (n = 7 for 0 µg/L; n = 6 for 2 µg/L; n = 7 for 5 µg/L and 10 µg/L; n = 2 for 20 µg/L). Linear model is significant (p < 0.05).

Supplementary Figures S4–S7 are presented to show the same biological endpoints as Figures 2–5 in the main text but based on nominal Pd concentrations. Given the substantial differences between nominal and measured values, these supplementary plots provide important context for interpreting the toxicological responses under both exposure metrics.


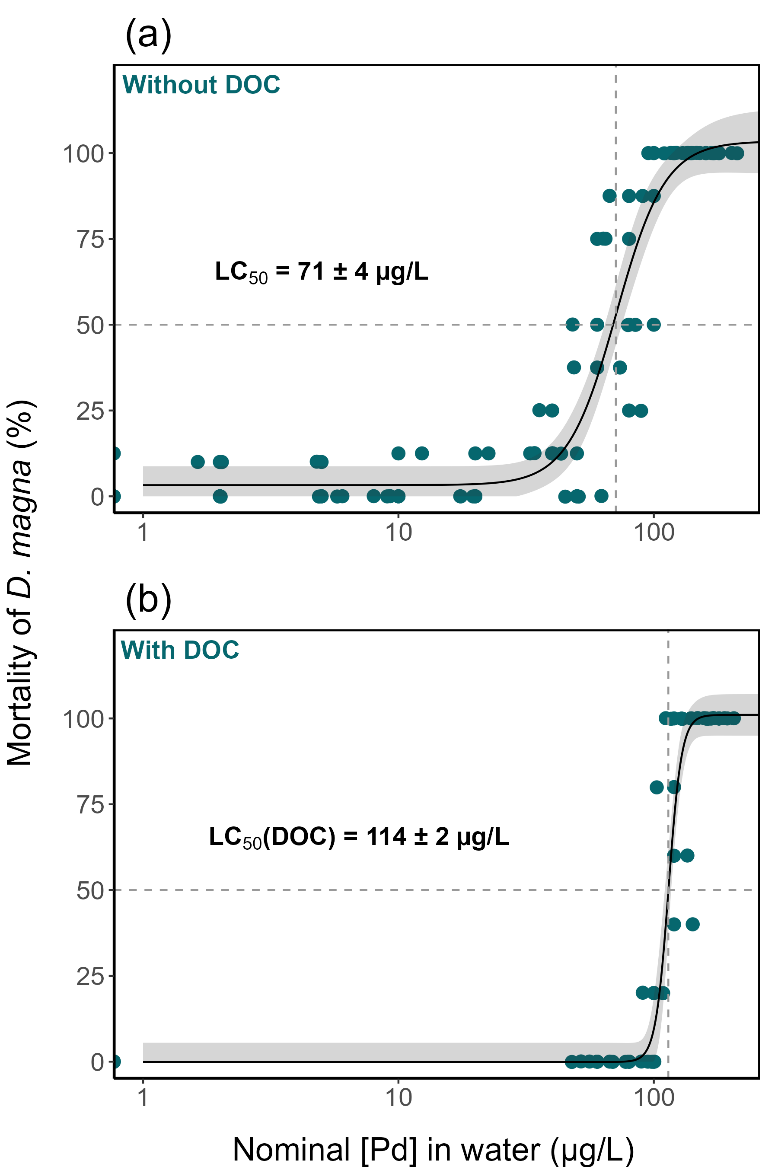


**Figure S4:** Concentration-response curves demonstrating the effect of Pd on the mortality of *D. magna* over a 48-hour exposure period in the presence (a) and absence (b) of dissolved organic carbon (DOC) (n = 4). Each dot represents the percentage of mortality within a replicate. In the presence of DOC (b), the measured DOC concentration was 0.8 mg C/L. The medium used was *Daphnia* culture water without M4 medium. Shaded areas represent the 95% confidence interval.


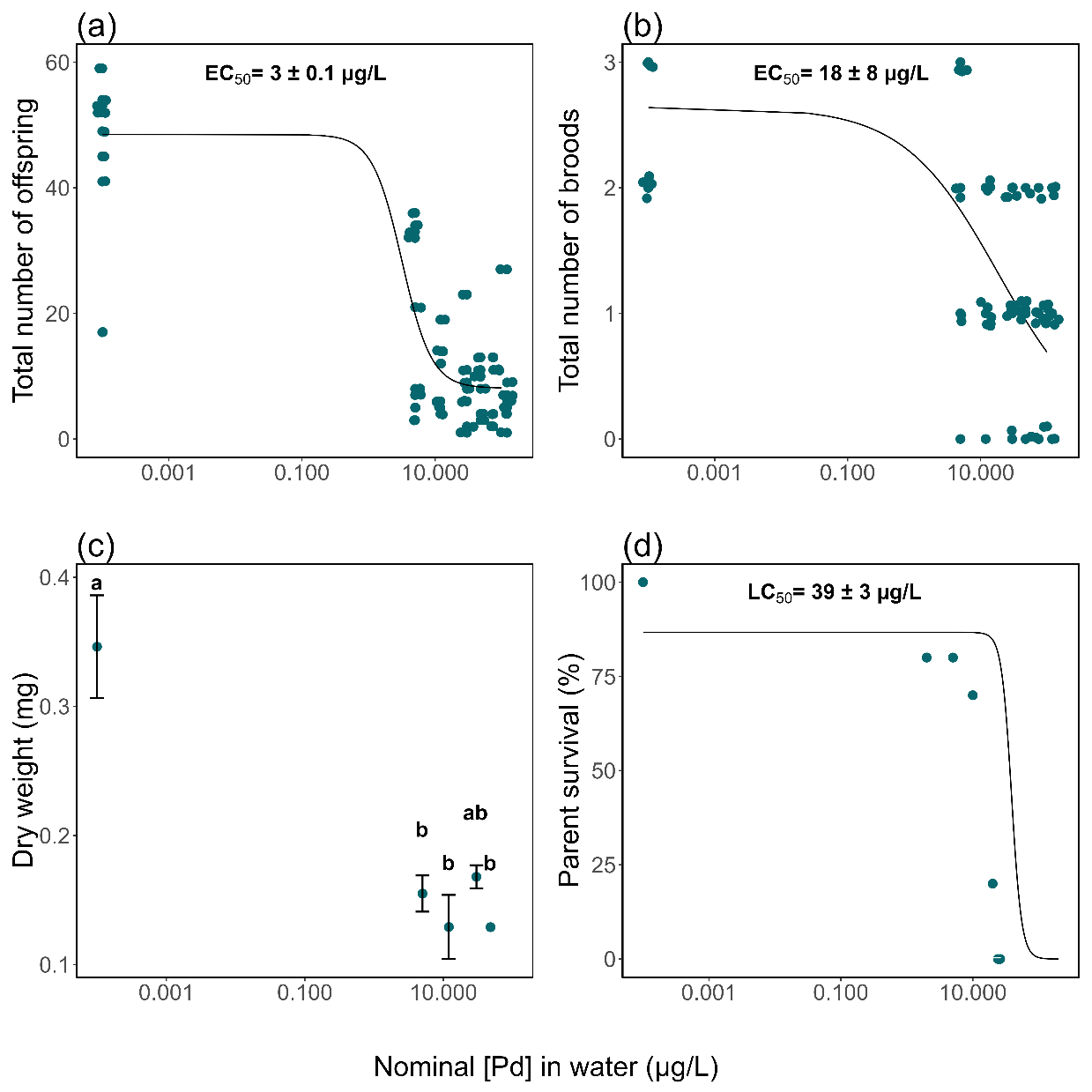


**Figure S5:** (a) Total number of offspring per *D. magna* (n=10 for 0 µg/L; n=9 for 2 and 5 µg/L; n=8 for 10µg/L; n=7 for 20 µg/L; n=8 for 24µg/L; n=5 for 26µg/L), (b) Total number of broods per *D. magna* (n=10), (c) Dry weight of the parent of *D. magna* (mg) (n=10 for 0µg/L; n=7 for 2µg/; n=8 for 5µg/L; n= 2 for 10µg/L; n=1 for 20µg/L), significant differences (p < 0.05) are indicated by distinct letters, (d) Survival of the parent of *D. magna* (n=10) as a function of measured Pd concentrations in water (Nominal [Pd] water, µg/L). The values represent mean ± standard error. Non-Linear regressions were fitted using the drm function in the drc package (R): a four-parameter log-logistic model (LL.4) for panel (a), and three-parameter log-logistic models (LL.3) for panels (b) and (d) (all models significant at p < 0.05).


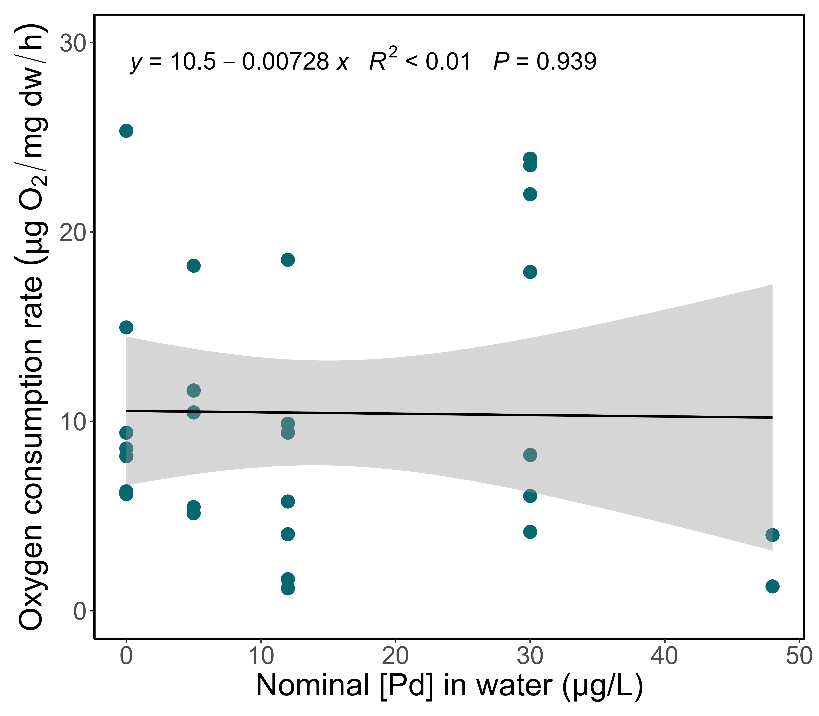


**Figure S6:** Oxygen consumption rate in adult *D. magna* after 15 days of exposure as a function of nominal Pd concentrations in water, standardized per mg dw of daphnid biomass (µg O_2_/mg dw/h) (n = 7 for 0 µg/L; n = 6 for 2 µg/L; n = 7 for 5 µg/L and 10 µg/L; n = 2 for 20 µg/L). Linear model is significant (p < 0.05).


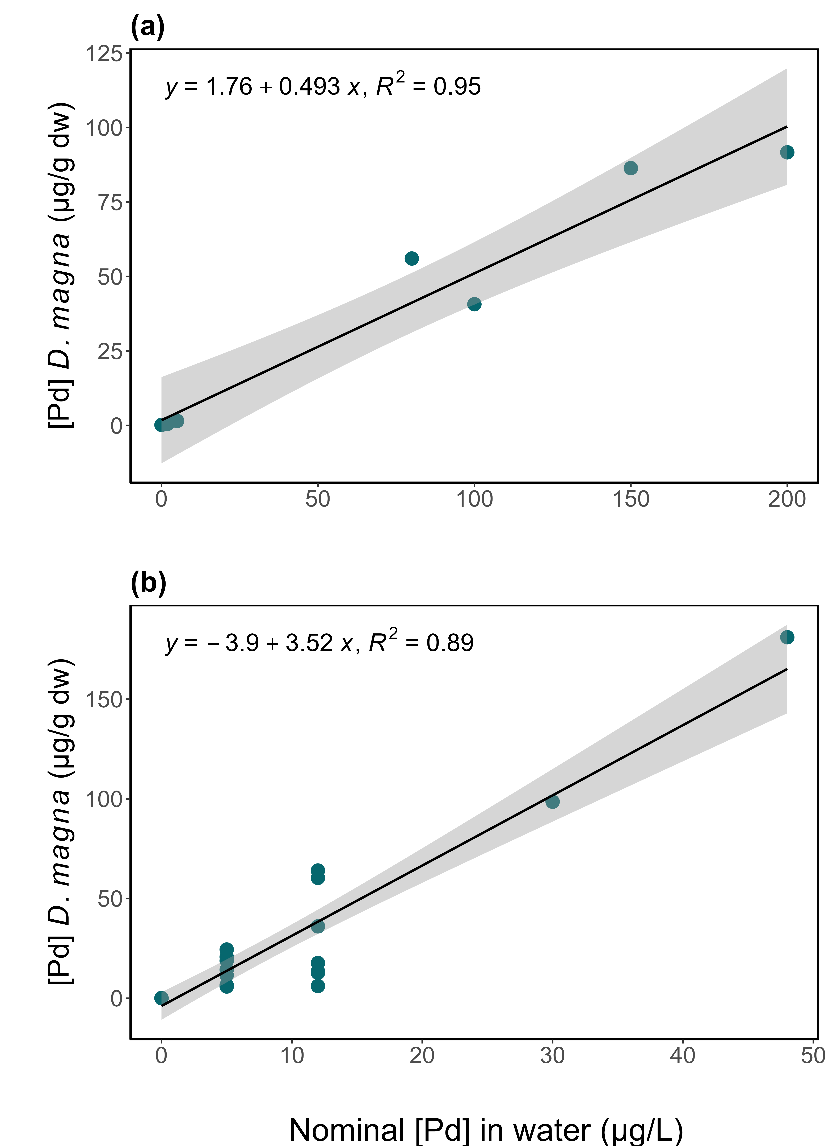


**Figure S7:** Pd bioaccumulation ([Pd] µg/g dry weight) in *D. magna* as a function of nominal Pd concentrations in water (Nominal [Pd] in water µg/L). Panel (a): acute exposure (48 h), each dot represents a pooled sample of 10 individuals per concentration. Panel (b): chronic exposure (15 days), each dot represents an individual surviving adult. Linear models are significant (p < 0.05).
